# EHS Guidelines on the Management of Primary Ventral and Incisional Hernias Under Emergency Conditions

**DOI:** 10.3389/jaws.2026.16228

**Published:** 2026-03-11

**Authors:** Cesare Stabilini, Alexis Theodorou, Maciej Pawlak, Stavros Antoniou, Frederik Berrevoet, Heather Bougard, Umberto Bracale, Sara Capoccia Giovannini, René Fortelny, Christine Gaarder, Miguel Angel Garcia-Urena, Katie Gilmore, Sergio Alejandro Gomez-Ochoa, Ferdinand Köckerling, Elisa Mäkäräinen, Salvador Morales-Conde, Francesca Pecchini, José Antonio Pereira Rodríguez, Andrea Carolina Quiroga-Centeno, Yohann Renard, Benoit Romain, Elena Schembari, Eva Deerenberg

**Affiliations:** 1 Departement of Integrated Surgical and Diagnostic Sciences, University of Genoa, Genoa, Italy; 2 University of Genoa, Genoa, Italy; 3 1st Department of Surgery, Hippocratio Hospital, University of Athens, Athens, Greece; 4 Department of General and Abdominal Wall Surgery, Golden Jubilee National University Hospital, Glasgow, United Kingdom; 5 School of Medicine Dentistry and Nursing, University of Glasgow, Glasgow, United Kingdom; 6 Medical School, European University Cyprus, Nicosia, Cyprus; 7 Department of General and HPB Surgery and Liver Transplantation, Ghent University Hospital, Ghent, Belgium; 8 Department of Surgery, New Somerset Hospital and University of Cape Town, Cape Town, South Africa; 9 Department of Gastroenterology, Endocrinology and Endoscopic Surgery, University Hospital of Naples, Federico II, Naples, Italy; 10 Medical Faculty, Sigmund Freud Private University Vienna, Vienna, Austria; 11 Institute of Clinical Medicine, University of Oslo, Oslo, Norway; 12 Department of Traumatology, Oslo University Hospital, University of Oslo, Oslo, Norway; 13 Grupo de Investigación de Pared Abdominal Compleja, Facultad de Medicina, Universidad Francisco de Vitoria, Hospital Universitario del Henares, Madrid, Spain; 14 Fundacion Cardiovascular de Colombia, Floridablanca, Colombia; 15 Department of General Internal Medicine and Psychosomatics, Heidelberg University Hospital, Heidelberg, Germany; 16 Hernia Center, Vivantes Humboldt-Hospital, Academic Teaching Hospital of Charité University Medicine, Berlin, Germany; 17 Gastrointestinal Surgery Department, Oulu University Hospital, Medical Research Center Oulu, Oulu, Finland; 18 Department of General and Digestive Surgery, University Hospital Virgen Macarena, University of Sevilla, Seville, Spain; 19 Unit of General and Digestive Surgery, Hospital Quirónsalud Sagrado Corazón, Seville, Spain; 20 Department of General Surgery, Emergency and New Technologies, Baggiovara General Hospital, AOU Modena, Modena, Italy; 21 Abdominal Wall Surgery Unit, Section of General Surgery, Department of General Surgery, Parc de Salut Mar, Hospital del Mar Medical Research Institute (IMIM), Barcelona, Spain; 22 Department of Surgery, Universidad Industrial de Santander, Bucamaranga, Colombia; 23 School of Translational Medicine, Medical Faculty Mannheim, Heidelberg University, Mannheim, Germany; 24 Reims Champagne-Ardennes, Department of General, Digestive and Endocrine Surgery, Robert Debré, University Hospital, Reims, France; 25 Department of Digestive Surgery, Centre Hospitalier Universitaire de Strasbourg, Strasbourg, France; 26 Department of Colorectal Surgery, Royal Devon and Exeter Hospital, Exeter, United Kingdom; 27 Department of Surgery, Franciscus Gasthuis en Vlietland, Rotterdam, Netherlands

**Keywords:** abdominal wall hernia, emergent repair, ventral hernia, incisional hernia, guidelines, mesh, laparoscopy

## Abstract

**Introduction:**

Emergent primary ventral or incisional hernias (PVIHs) are a common cause of surgical admission, leading to significantly higher rates of morbidity and mortality compared to elective hernia repairs. Despite this, management varies widely due to a lack of evidence-based consensus. This article presents the new European Hernia Society (EHS) guidelines for the emergency treatment of adult patients with PVIH.

**Material and Methods:**

This project was developed by the EHS Science Committee following AGREE-S, GRADE, and GIN standards. A guideline panel, composed of general and emergency surgeons along with patient partners, formulated seven key health questions addressing the surgical approach, mesh type and placement, and the management of defects of varying sizes and contamination levels to support general surgeons in their decision-making process. A systematic review was conducted, and recommendations were developed using a formal evidence-to-decision framework, ensuring consensus was reached on all recommendations.

**Results:**

The guidelines expert panel provides recommendations for several clinical scenarios. For defects amenable to direct closure, mesh-based repair is suggested over primary suture repair, regardless of the contamination grade. Furthermore, a laparoscopic approach with intraperitoneal mesh, an open approach with onlay mesh placement, and the use of large-pore synthetic meshes are recommended. For large defects, not amenable to closure, a staged approach that avoids immediate mesh-based repair is suggested.

**Conclusion:**

Adherence to these guidelines can help standardise the management of emergent PVIHs, potentially improving patient outcomes. The recommendations advocate for a “damage control” mindset, prioritising physiological stability over immediate definitive reconstruction. Further research is needed to address gaps in the current literature, particularly with regard to long-term recurrence rates and the specific protocols for managing these complex cases.

## Introduction

Primary Ventral and incisional hernias (PVIHs) represent conditions that negatively impact patients’ quality of life and require surgical repair [1, 2]. The emergency presentation of abdominal wall and groin hernias is one of the most severe complications, representing a common reason for emergency surgical admission and accounting for around 25% of all hernia repairs [3, 4]. Trends in emergent incisional hernia repair have remained relatively stable among older women, at 24.9 and 23.5 per 100,000 person-years in 2001 and 2010, respectively. However, rates of emergent incisional hernia repair among older men are reported to have risen significantly, from 7.8 to 32.0 per 100,000 person-years from 2001 to 2010, respectively. The outcomes of emergent incisional hernia repair are worse than those of elective repair; a tenfold increase in mortality has been reported, along with increased morbidity rates [5, 6]. A recent scoping review performed by our group showed that pooled proportions of surgical site infections (SSIs), mortality, any complications, and the risk of reoperation were 12%, 4%,31%, and 8%, respectively [7].

The emergent presentation of a ventral or incisional hernia can give rise to different clinical scenarios, ranging from pain alone to irreducibility, bowel obstruction, strangulation and, ultimately, peritonitis secondary to perforation; these scenarios represent a spectrum that is time-dependent and severely impacts subsequent treatment. General surgeons facing this situation are compelled to make difficult decisions concerning the surgical approach (e.g., open vs. laparoscopic), the choice of mesh (synthetic, biological, or biosynthetic), the type of repair, and the management of contaminated fields or bowel dilation.

Several factors further complicate decision-making in these scenarios. The lack of adequate patient prehabilitation, coupled with the difficulty of achieving true shared decision-making due to time constraints, significantly impacts treatment choices [8–10]. Furthermore, specialists in abdominal wall surgery are not always readily available for complex defects, meaning that the majority of management is the responsibility of general surgeons, who often have limited expertise in this specific field.

The World Society of Emergency Surgeons has previously issued guidelines for managing emergent patients with these conditions, with their latest iteration in 2020 focusing primarily on wound contamination status, encompassing every type of abdominal wall defect (inguinal, primary ventral, and incisional) [11].

Complementing this, the European Hernia Society has decided to compile guidelines focusing on abdominal wall hernias that are different from inguinal hernias [12, 13], based on the belief that the clinical presentation of emergent primary ventral and incisional hernias may necessitate a prioritisation of surgical objectives, whereby the hernia itself may not be the primary focus of treatment, but merely the *primum movens* *.* This guideline project, specifically targeted at general surgeons without a special interest in abdominal wall surgery, is devoted to developing an intraoperative decision-making aid (algorithm) based on defect characteristics, contamination and patient stability.

This document details these recommendations, outlines the framework used to develop these guidelines, and provides the supporting scientific data with their critical appraisals.

## Materials and Methods

This project was developed by the Science Committee of the European Hernia Society. The present guideline follows AGREE-S [14, 15], GRADE [16] and Guidelines International Network (GIN) development and reporting standards. [17–19]. An AGREE-S reporting checklist is provided in Supplementary Material 1.

The online platform GRADEpro GDT (GRADEpro Guideline Development Tool, McMaster University and Evidence Prime, 2025,^1^) was used to generate evidence tables and summarise evidence-to-decision considerations. The current guidelines aim to provide general and emergency surgeons with practice recommendations, supported by the best available evidence, for the emergency treatment of adult patients presenting with primary ventral and incisional hernias.

### Steering Group

The steering group consisted of four general and abdominal wall surgeons with a specific interest in hernia surgery (CS, MP, ED, and AT), members of the EHS Board and/or experts in guideline development and evidence synthesis. They reported no financial or intellectual conflicts of interest, as per GIN standards [20].

One of the members of the steering committee (CS) is a certified guideline methodologist in training through the INGUIDE programme [17] and led the guideline under the supervision of a certified guideline methodologist (SAA - INGUIDE certificate number 2022-L3-V1- 00014).

### Guideline Panel

The panel composition (Supplementary Material 2) aimed to ensure representation of all genders, international diversity, and both academic and non-academic affiliations. All members of the panel received training and certification through the INGUIDE programme (level 1 certification).

### Evidence Review Teams

Two teams executed the systematic search: the clinical review team (ED, AT, ACQ, SCG, KG, UB, BR, and YR) handled article retrieval and outcome reporting, while the EtD team (FP, SCG, and ES) identified framework context items such as equity, feasibility, and patient values.

### Project Development and Meetings

The project, initiated in November 2023 on behalf of the European Hernia Society (EHS), was conceived within the framework of the ENGINE project for guideline production and updates [21].

The inaugural kick-off meeting was held in May 2024 in Prague, during the European Hernia Society’s annual conference.

In January 2025, a face-to-face meeting was held in Genoa, where the summarised evidence and provisional recommendations were discussed and then finalised during a subsequent online meeting in March 2025.

The results, recommendations, and treatment algorithm were presented during a plenary session at the EHS annual conference in Paris in June 2025.

### Health Questions

These guidelines (GLs) address the following health questions, referring to the Centers for Disease Control and Prevention (CDC) classification for wound contamination.

- KQ 1 – Should mesh-based repair vs. tissue repair be used for the emergency treatment of a PVIH hernia in CDC I, stable patients with a defect amenable to direct closure?
- KQ 2 – Should mesh-based repair vs. tissue repair be used for the emergency treatment of a PVIH in CDC ≥II, stable patients with a defect amenable to direct closure?
- KQ 3 – Should mesh-based repair vs. no repair be used for the emergency treatment of a PVIH in CDC I, stable patients with a defect not amenable to direct closure?
- KQ 4 – Should mesh-based repair vs. no repair be used for the emergency treatment of a PVIH in CDC ≥II, stable patients with a defect not amenable to direct closure?
- KQ 4a- Should closure of the abdominal cavity vs. open abdomen be used for the emergency treatment of a PVIH in CDC ≥II, stable patients with a defect not amenable to direct closure?
- KQ 5 – Should retromuscular mesh placement vs. other mesh placement positions be used for the emergency repair of acutely complicated PVIH?
- KQ 6 – Should synthetic permanent mesh vs. other mesh types be used for the emergency treatment of adult patients with acutely complicated PVIH?
- KQ 7 – Should a laparoscopic vs. open approach be used for the emergency treatment of stable adult patients with acutely complicated PVIH?

The panel acknowledges that pooling primary and incisional hernias represents a methodological limitation with regard to the different features of these defect types [22], but this grouping was dictated by the inherent heterogeneity of the defects in the relevant literature and the lack of sufficient subgroup data in the existing studies.

This guideline specifically refers to adult patients with acutely complicated abdominal wall hernias (primary ventral or incisional) who are deemed fit for surgery and are in a stable condition. The hernia contents can include preperitoneal fat, omentum, or viscera, which can become acutely irreducible and may be associated with obstruction, strangulation or perforation.

Open abdomen (OA), burst abdomen (BA), and acutely complicated parastomal hernias are outside the scope of the present document. For optimal management in such scenarios, readers should refer to the ‘EHS clinical guidelines on the management of the abdominal wall in the context of the open or burst abdomen’ and the European Hernia Society guidelines on the prevention and treatment of parastomal hernias [23, 24].

### Targeted Users

This project aims to provide a decision tool for general, abdominal, trauma and emergency surgeons facing this clinical scenario in cases where a specific abdominal wall subspecialist is unavailable.

A specific patient-friendly version of the present GLs will be made available and developed in accordance with the EHS Secretary for Publications.

### Definitions

The following section refers to the standards and nomenclature adopted for the present guidelines.

#### Acutely Complicated PVIHs

Using the same terminology adopted in the inguinal hernia guidelines [13], the following terms are proposed:

- Acutely irreducible hernia—an abdominal wall defect in which the contents cannot be reduced on physical examination, but were previously reducible prior to the acute onset of symptoms.
- Chronically irreducible hernia—an abdominal wall defect in which the contents cannot be reduced on physical examination, which is of long standing and is not associated with the sudden onset of new symptoms.
- Strangulated hernia—A strangulated abdominal wall defect is an irreducible hernia where the constriction at the neck of the hernia sac compromises blood flow to its contents. This ischaemia can rapidly lead to tissue infarction and gangrene. It can only be described as such after the diagnosis is confirmed by preoperative imaging or intraoperative findings [13].

#### Clinical Stability

Stable patients are those presenting without impaired vital parameters, in particular those not determining a systemic inflammatory response syndrome (SIRS), sepsis or septic shock [25]. We encourage assessment of the patient’s condition using SIRS or quick Sequential Organ Failure Assessment (qSOFA) criteria to define clinical stability [26]:

qSOFA uses three components for assessment:

- Systolic blood pressure <100 mm Hg
- Highest respiratory rate >21
- Lowest Glasgow coma score <15
- Patients referred to in the present guidelines are those with none of these features.

#### Defects Amenable/Not Amenable to Closure

According to the European Hernia Society guidelines on incisional hernia management [27], a defect is considered not amenable to direct closure if its repair would necessitate any form of myofascial release to approximate the defect edges. Based on preoperative CT imaging, these defects typically exhibit one or more of the following physical characteristics: a width exceeding 8 cm, an area greater than 164 cm^2^, a rectus-to-defect ratio of less than 1.34, or a component separation index (CSI) greater than 0.146 [28]. The expert panel also suggests that Loss of Domain (LOD), calculated using the Sabbagh method, may serve as a decisive factor in determining the feasibility of a tension-free abdominal closure [29].

#### Grade of Contamination

Wound contamination is classified according to the Centers for Disease Control and Prevention (CDC):

- Class I-Clean These types of wounds are not infected, do not exhibit any signs of inflammation, and are typically closed.
- Class II- clean contaminated These wounds have a low level of contamination. They involve entry into the respiratory, alimentary, genital, or urinary tracts, but only under controlled circumstances.
- Class III- contaminated These typically result from a breach in sterile techniques or leakage from the gastrointestinal tract. Incisions resulting from acute or non-purulent inflammation are also considered Class III wounds.
- Class IV-dirty These are considered infected due to the inadequate treatment of traumatic wounds, gross purulence, and evident infection. When tissues lose vitality, this can lead to Class IV wounds. This is often caused by surgery or by microorganisms found in perforated organs [30].

#### Source Control

The term “Source control” is used to identify the set of all physiological/pharmacological/interventional measures adopted to control a focus of infection, to modify factors in the infectious milieu that promote microbial growth or impair host antimicrobial defences, and to allow the organism to recover homeostasis or at least a sort of “physiological equilibrium” [31].

#### Abdominal Wall Management Techniques

- Primary Fascial Closure is defined as the closure of the defect by suturing the aponeurotic/muscular edges of the defect.
- Mesh-Based Repair is defined as the use of any mesh material (biologic, synthetic, or biosynthetic) for the purpose of providing an abdominal wall repair. This can be achieved by:
  ○ Any form of bridging
  ○ Mesh augmentation after Primary Fascial Closure (PFC); the description of the possible plane for mesh placement follows the principles of the International Classification of Abdominal Wall Planes (ICAP classification) [32].
- Closure of the abdominal cavity (CAC or planned incisional hernia) is defined as suturing of the hernia sac and/or the skin (it can be reinforced with polyglactin-910 woven absorbable mesh) as a bailout measure, with no intention of providing any abdominal wall repair [33].
- Open Abdomen (OA) is a procedure in which the fascial edges of the abdomen are intentionally left unapproximated (laparostomy) with the exposed abdominal contents protected by temporary coverage. OA, both in trauma and non-trauma patients, is used to decompress the abdomen in the presence of severe peritonitis or sepsis caused by intestinal perforation, vascular emergencies, or severe pancreatitis. This technique helps prevent subsequent abdominal compartment syndrome and facilitates a surgical second look [23, 34].

Both CAC and OA are included in Temporary abdominal wall closure (TAC) strategies, along with others not included in the present guidelines.

### ICAP Planes

- Onlay: The mesh is positioned superficial to the anterior rectus sheath and external oblique aponeurosis, lying deep to the subcutaneous tissue.
- Inlay: The mesh is secured to the margins of the fascial defect without overlapping the surrounding native tissue.
- Retrorectus or retromuscular: The mesh is placed posterior to the rectus abdominis muscle and anterior to the posterior rectus sheath, or posterior to the transverse abdominis muscle.
- Intraperitoneal: The mesh is situated posterior to the peritoneum, within the abdominal cavity.
- Bridging: While not referring to an individual anatomical plane, “bridging” describes a reconstructive technique in which fascial closure of the defect is not performed.

This concept can be applied as a clarification to any of the aforementioned planes to indicate that the mesh spans the defect without direct approximation of the native fascia [32].

### Protocol and Amendments

A protocol was developed by the steering group and published in JAWS [35]. The guidelines’ questions and outcomes were refined in collaboration with the guidelines panel members.

To better align with the specific scenarios being analysed, the comparison terms for KQ3 and KQ4 have been modified from “mesh” and “sutures” to “repair” versus “no repair”. This change prevents misunderstandings about repair techniques that were judged to be not relevant to the current analysis by the panel, because they are impossible to accomplish (direct suturing) or possibly detrimental for the patients (component separation without mesh).

### Rating the Importance of Outcomes and Setting Minimal Important Differences

The importance of outcomes was rated by panel members using the GRADE scale [36].

Outcomes were classified by the steering group into each of the three categories (not important, important, critical) under consideration of panel members’ ratings. The panel considered the importance of outcomes and thresholds as results of the survey on outcomes and differences available in Supplementary Material 3. According to the previously published scoping review [7], the outcome “Quality of Life” could not be prioritized since it has never been reported in papers.

### Search Strategy

A practical decision was made to conduct a single comprehensive search covering all KQs due to the little available literature on the topic.

The search included studies published on PubMed and SCOPUS between 1 January 2000 and 1 November 2024 to capture contemporary evidence, with no language restrictions adopted. Moreover, series with fewer than 20 patients, systematic reviews and meta-analyses, case reports, editorials and letters were excluded from the search. The research was updated in November 2025 to include all recently published articles, using the same search strings. The search syntaxes are provided in Supplementary Material 4.

### Study Selection and Data Extraction

The evidence review team (ED, AT, AC, SC, KG, UB, BR, and YR) performed record screening using the ASreview platform [37]. Reviewers were blinded to each other’s judgements, and the senior author (CS) resolved any disagreements after unblinding. The same reviewers, in collaboration with the methodologist, selected articles based on full-text screening.

The overarching inclusion criterion was adult patients with abdominal wall defects presenting under emergency conditions, but specific inclusion and exclusion criteria were adopted and are provided in Supplementary Material 4.

After the definition of relevant outcomes, an Excel spreadsheet was prepared to allow uniform data collection (Supplementary Material 5).

### Statistical Analysis

All statistical analyses were conducted independently by a designated statistician, with no involvement from the steering group or panel members. The analysis plan was specified *a priori* in the guideline protocol.

For each KQ, where data were available from two or more studies, a meta-analysis was performed. Given the anticipated clinical and methodological heterogeneity across studies (e.g., variations in patient populations, specific surgical techniques, and study designs), a random-effects model using the DerSimonian and Laird method was employed for all meta-analyses. This approach provides a more conservative estimate of the treatment effect by incorporating both within-study and between-study variance.

For dichotomous outcomes such as mortality, surgical site infection, reoperation, and recurrence, Odds Ratios (ORs) with 95% Confidence Intervals (CIs) were calculated. The selection of ORs as a common measure of effect was derived from the inclusion of case-control studies in the meta-analysis, as in this study type, the incidence of outcomes is not directly estimable, and risk ratios (RRs) cannot be calculated. When zero events occurred in one arm, a continuity correction of 0.5 was applied to all cells in the 2 × 2 table. Statistical heterogeneity was assessed using both the Cochran’s Q test (with a p-value <0.10 indicating significant heterogeneity) and the I^2^ statistic. The I^2^ statistic was interpreted as follows: <40% indicates low heterogeneity, 40%–75% indicates moderate heterogeneity, and >75% indicates substantial heterogeneity. Subgroup analyses were planned to investigate potential sources of heterogeneity, such as differences between primary and incisional hernias, and varying degrees of contamination. However, due to limitations in the data reported in the primary studies, these analyses were not feasible. Similarly, an assessment of publication bias using funnel plots and Egger’s test was planned. However, this was not conducted for any of the KQs, as each meta-analysis included fewer than 10 studies, a threshold below which these tests are considered to have low power and can produce misleading results. All statistical analyses were performed using Cochrane RevMan Web. A two-sided p-value <0.05 was considered statistically significant for all analyses except for the assessment of heterogeneity, for which a p-value <0.10 was used.

### Risk of Bias

RoB-2 and ROBINS-I [38, 39] were used to assess the risk of bias in randomised controlled trials and cohort studies with a comparative arm, respectively. All statistical analyses were performed independently by the statisticians’ group, with no involvement from the steering group or panel members.

### Certainty of Evidence

The certainty of evidence was determined by the risk of bias across studies, incoherence, indirectness, imprecision, publication bias, and other parameters [40].

Minimal important differences, which were determined in advance through a survey of panel members, were used to inform the judgements about precision and coherence.

A formal anonymous online vote was carried out to finalise the judgements during the in-person and remote meetings. A total of three meetings were held: one in October 2024 (online), one in January 2025 in Genoa (in person), and one in March 2025 (online). Based on the evidence-to-decision framework, the panel anonymously voted on the strength and the direction of the recommendation through Google Forms.

### Evidence-To-Decision Framework and Recommendations

We used the evidence-to-decision framework to develop recommendations by considering the following factors [41]:

- Anticipated benefits and harms of the intervention
- The certainty of evidence
- Values and preferences of patients and healthcare providers
- Resources required
- Acceptability of the intervention
- Feasibility of implementing the intervention
- Equity considerations

External advisors contributed to discussions but did not participate in judgements on the evidence-to-decision domains. Following the consensus meeting, panel members completed an anonymous online vote on the direction and strength of each recommendation and were invited to propose modifications in accordance with the GRADE methodology [42].

We considered consensus to be agreement among >70% of panel members if, after exhaustive deliberations, a unanimous consensus was not achieved.

### Direction and Strength of Recommendations

- Strong Recommendation: the panel is highly confident that the desirable effects clearly outweigh the undesirable effects for most, if not all, patients. This means that the majority of informed individuals would choose the recommended action.
- Conditional Recommendation: the panel is less confident that the desirable effects clearly outweigh the undesirable effects. This is often because the certainty of evidence is low, the benefits and harms are closely balanced, or patient values and preferences vary significantly. Therefore, patient choices may differ based on their individual values and circumstances.

## Results

The systematic review identified one Randomised Controlled Trial (RCT), four prospective studies, and nineteen retrospective studies that contributed to the evidence base for all Key Questions. Details and references are provided in Supplementary Material 6. Records excluded during the first and second levels of screening are detailed in the PRISMA 2020 Flow Diagram, provided in Supplementary Material 7.

The risk of Bias Assessment at the outcome level is available in Supplementary Material 8.

It was not feasible to perform subgroup analyses of primary ventral and incisional hernias due to limitations in data extraction. Similarly, separate analyses for different CDC wound classes (II-III or IV) were not possible for KQ2 and KQ4. Furthermore, comparisons between biosynthetic versus synthetic meshes could not be made due to a lack of comparative studies.

The evidence tables and forest plots supporting the recommendations are presented in Supplementary Material 9–15.

### Recommendations

The present recommendations are intended as a tool for general, abdominal, trauma and emergency surgeons, who are not specialists in abdominal wall surgery.

**FIGURE 1:**
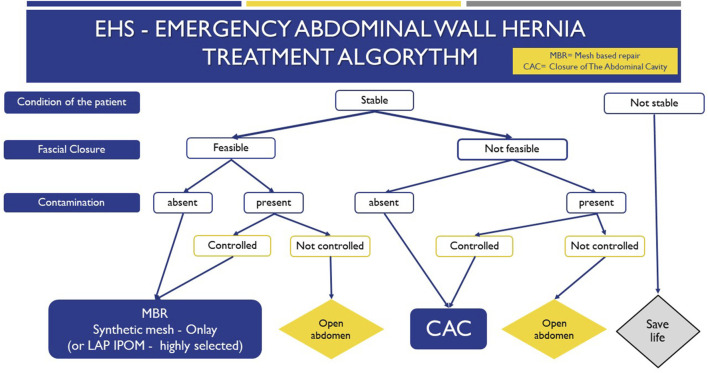
EHS emergency ventral and incisional hernia management algorithm.

Tables 1–8 present the final recommendations, the evidence supporting them and an explanatory text detailing the additional evidence and panel considerations. Figure 1 (EHS - Emergency Ventral Hernia Treatment Algorithm) illustrates the recommended algorithm for emergency PVIH management.

**TABLE 1:** KQ1: Should mesh-based repair vs. tissue repair be used for the emergency treatment of a PVIH hernia in CDC I, stable patients with a defect amenable to direct closure?

Recommendation: In stable patients undergoing emergency treatment for a PVIH with CDC class I wounds and defects amenable to direct closure, we suggest a mesh-based repair rather than primary fascial closure		
Conditional recommendation in favour of mesh repair-low certainty of evidence [43–52]		
Clinical outcomes		
Recurrence	OR 0.47; 95% CI 0.31–0.72 (8 studies-n = 45,037)	Favour mesh repair
SSI	OR 0.41; 95% CI 0.15–1.15 (4 studies-n = 399)	No difference
Mortality	OR 0.38; 95% CI 0.33–0.45 (2 studies- n = 43,861)	Favour mesh repair
Evidence for decision framework considerations (nomenclature for judgement on effect is based on GRADE methodology- please see https://gdt.gradepro.org/app/handbook/handbook.html):		
Desirable effects	Research evidence	Judgement on effect
	The panel considered that mesh is associated with moderate benefits in terms of hernia recurrence	Moderate
	Additional considerations	
	None of the studies reported data on QoL; only one of them used a generic questionnaire to assess QoL, but this was not focused on evaluating the difference between mesh and tissue repair. The panel agreed that the recurrence rate could be used as an indirect tool to assess QoL; consequently, a reduced recurrence rate after mesh repair would have a positive impact on it [50, 53]	
Undesirable effects	Research evidence	Trivial
	Major morbidity, reoperation and SSI appear similar for both approaches and do not appear to be altered by mesh implantation	
	The difference in mortality encountered in the analysed articles is considered flawed due to methodological bias, particularly with regard to patient selection	
	Additional considerations	
	Some mesh-related complications (erosion, adhesions) can be anticipated, but this largely depends on the placement technique and materials used, and their occurrence is limited	
Certainty of evidence	The certainty of evidence across the body of literature reviewed was considered to be low to very low across comparisons due to limited evidence regarding critical outcomes; sensitivity analyses were not possible	Very low
Balance of effects	The panel evaluated that, when comparing equal levels of morbidity, prosthetic repair for defects in CDC 1 provides a significant advantage in terms of long-term repair, surpassing simple direct repair	Possibly favours mesh repair
Values	Research evidence	Possibly important uncertainty or variability
	A scoping search of the literature did not identify relevant evidence regarding patient values and preferences	
	Additional considerations	
	No important variability is expected concerning mortality outcomes	
	Important variability in patient preferences with regard to the other outcomes prioritised is expected. The patient representative declared that they usually rely on the choices of the surgeon; nevertheless, they would favour a strategy based on immediate repair to avoid repeated procedures and related risks	
Resources	Research evidence	Moderate savings
	No evidence regarding the resources required was identified	
	No evidence concerning cost-effectiveness to support decision-making was identified	
	Additional Considerations	
	Only indirect considerations could be made: the use of polypropylene mesh currently adds costs to the procedure that are negligible	
	A single-stage definitive repair (with mesh) requires one admission, while a postponed mesh repair would require more procedures, a new hospitalisation, and a possible longer in-hospital stay with possible increased costs	
Certainty of evidence of required resources	Research evidence	Very low
	The certainty of the evidence regarding the required resources is very low. No direct comparisons were made, and only indirect conclusions could be drawn and discussed within the panel	
Equity	Research evidence	Likely to have no impact
	No evidence regarding equity was identified	
	Additional considerations	
	No threats were identified in the discussion with the panel	
Acceptability	Research evidence	Variations in acceptability can be anticipated
	No evidence concerning acceptability from patients has been found	
	Among surgeons, there are discrepancies in panel opinions, while according to Crepaz et al, 65.4% of the participating surgeons considered mesh positioning adequate [54]. In the MASH survey, 74% of surgeons favoured mesh implants in clean cases under emergency conditions (70% permanent synthetic) [55]	
	Additional considerations	
	The panel agreed that both suture and mesh repair were likely to be acceptable to surgeons, but there may be variations in patient preferences regarding the use of mesh (with both positive and negative perceptions)	
	Patient advocates said that they would trust their surgeon with the selection of the best approach	
Feasibility	Research evidence	Yes
	No evidence regarding feasibility was identified	
	Additional considerations	
	The panel suggested that there is likely to be minimal variation in the ability to perform the alternatives. The intervention should already be part of common practice and hence feasible to implement	

**TABLE 2:** KQ2: Should mesh-based repair vs. tissue repair be used for the emergency treatment of a PVIH in CDC ≥II, stable patients with a defect amenable to direct closure?

Recommendation		
In stable patients undergoing emergency treatment for a PVIH with CDC class ≥II wounds and defects amenable to direct closure, once adequate source control has been achieved, we suggest mesh-based repair rather than primary fascial closure		
Conditional recommendation in favour of mesh repair-Very low certainty of evidence [46, 56–58]		
Clinical outcomes		
Recurrence	OR 0.24; 95% CI 0.09–0.612 (studies- n = 371)	Favour mesh
SSI	OR 1.17; 95% CI 0.93–1.47 (3 studies- n = 2,820)	No difference
Evidence for decision framework considerations (nomenclature for judgement on effect is based on GRADE methodology- please see https://gdt.gradepro.org/app/handbook/handbook.html):		
Desirable effects	Research evidence	Judgement on effect
	The panel concluded that mesh is associated with moderate benefits in terms of hernia recurrence (threshold 5% - value 9.3%)	Moderate
Undesirable effects	Research evidence	Trivial
	The effects on the available prioritised outcomes (mortality, SSI) appear to be similar for both interventions	
	Some mesh-related complications (erosion, adhesions) can be anticipated, but largely depend on the placement technique and materials used, and the related risk is small	
	Additional considerations	
	The range of clinical scenarios that can be included in CDC >2 ranges from resection anastomosis to overt faecal peritonitis; accordingly, undertaking the repair should always be done when source control of infection is achieved, and definitive repair is considered	
Certainty of evidence	The certainty of evidence was very low; there was limited evidence regarding critical outcomes. Sensitivity analyses were not possible	Very low
Balance of effects	Research evidence	Likely to favour mesh repair
	The panel considered that there is a net benefit in favour of the intervention, particularly in terms of a lower likelihood of hernia recurrence and a similar likelihood of SSIs	
Preferences and values	Research evidence	Possibly important uncertainty or variability
	A scoping search of the literature did not identify any relevant evidence concerning patient values and preferences	
	Additional considerations	
	No important variability is expected concerning mortality outcomes	
	Important variability in patient preferences with regard to the other outcomes prioritised	
	The patient representative declared that they usually rely on the choices of the surgeon; nevertheless, they would favour a strategy based on an immediate repair to avoid repeated procedures and related risks	
Resources	Research evidence	Moderate savings
	The cost of a large-pore synthetic mesh is negligible	
	Additional considerations	
	A single-stage definitive repair (with mesh) requires one admission, while a postponed mesh repair would require more procedures, a new hospitalisation, and a possible longer in-hospital stay with possible increased costs	
	No evidence concerning cost-effectiveness to support decision-making was identified	
Certainty of evidence of required resources	Research evidence	Very low
	The certainty of evidence regarding required resources is very low. No direct comparisons were made, and only indirect conclusions could be drawn and discussed within the panel	
Equity	Research evidence	Possibly no impact
	No evidence regarding equity was identified	
	Additional considerations	
	No threats were identified in the discussion with the panel	
Acceptability	Research evidence	Variations in acceptability can be anticipated
	No evidence concerning acceptability from patients was found. Among surgeons, the major concern regarding mesh repair in CDC ≥2 is the risk of mesh-related adverse events (infection and explant) and there are discrepancies in authors’ opinions; the survey by Mariette et al showed that 85% of respondents preferred direct suturing without and with component separation in contaminated fields [59], while according to Crepaz et al 65.4% of the participating surgeons considered mesh positioning to be adequate [54]	
	Additional considerations	
	The panel agreed that both suture and mesh repair were likely to be acceptable to surgeons, but there may be variation in patient preferences regarding the use of mesh (with both positive and negative perceptions)	
	Patient advocates said that they would trust their surgeon to select the best approach	
Feasibility	Research evidence	Yes
	No evidence regarding feasibility was identified	
	Additional considerations	
	The panel suggested that there is likely to be minimal variation in the ability to perform the alternatives. The intervention should already be part of common practice and hence feasible to implement	

**TABLE 3:** KQ3: Should mesh-based repair vs. no repair be used for the emergency treatment of PVIH in CDC I, stable patients with a defect not amenable to direct closure?

Recommendation		
In stable adult patients with acutely complicated PVIH with CDC class I wounds and defects not amenable to direct closure, we suggest deferring definitive abdominal wall reconstruction rather than performing immediate reconstructive repair. Explanatory note: Closure of the abdominal cavity may be obtained through skin closure, sac closure, inlay placement of absorbable mesh, or mesh bridging		
Conditional recommendation against repair -Very low certainty of evidence [34]		
Clinical outcomes		
Recurrence	OR 0.07; 95% CI 0.01–0.42 (1 study-n = 40)	Despite data favouring repair for recurrence, the high risk of selection bias suggests that deferring reconstruction remains the more cautious approach in an emergency context
Evidence for decision framework considerations (nomenclature for judgement on effect is based on GRADE methodology- please see https://gdt.gradepro.org/app/handbook/handbook.html):		
Desirable effects	Research evidence	Judgement on effect
	Concomitant repair of the abdominal wall is feasible in experienced hands in highly selected cases, with advantages for the patient	Moderate
	Additional considerations	
	Addressing small bowel obstruction and liberating incarcerated hernia sac contents from adhesions are crucial steps to avert the progression to life-threatening complications (e.g., strangulation and perforation)	
Undesirable effects	Research evidence	Large
	Danish database: In the emergency setting, there is a tenfold increased risk of complications after retromuscular mesh placement vs. onlay (OR = 10.12, 95% CI = 1.81–56.68, p = 0.008), with increasing rates observed in cases of larger defects and advanced comorbidities [60]. AHSQC database: TAR under emergency conditions: Increased risk of adverse events (wound morbidity - SSO 25.4%, SSI 13.6%, SSOPI 13.6%; reoperation within 30 days: 5.1%, any complication: 37.3%) [61]	
	Deferring abdominal wall repair determines a possible increase in the width of the defect [62]	
Certainty of evidence	Research evidence	Very low
	The certainty of evidence across the body of literature reviewed was considered to be low to very low	
Balance of effect	Research evidence	Likely to favour the comparison
	The increased risk of serious adverse events in cases of abdominal wall reconstruction performed in an obstructed emergent setting outweighs the possible advantages represented by a single procedure that addresses both bowel continuity and abdominal wall continence issues	
Values	Research evidence	Possibly important uncertainty or variability
	A scoping search of the literature did not identify any relevant evidence with regard to patient values and preferences	
	Additional considerations	
	No important variability is expected with regard to mortality outcomes	
	Important variability in patient preferences with regard to the other outcomes prioritised is expected	
	The patient representative declared that they usually rely on the choices of the surgeon; nevertheless, they would favour a strategy based on immediate repair to avoid repeated procedures and related risks	
Resources	Research evidence	Moderate savings
	The resources required for simultaneous abdominal wall reconstruction or no repair can vary significantly, as can the costs associated with complications	
Certainty of evidence of required resources	Research evidence	Very low
	The certainty of evidence regarding the required resources is very low. No direct comparisons were made, and only indirect conclusions could be drawn and discussed within the panel	
Equity	Research evidence	Probably no impact
	No evidence regarding equity was identified	
	Additional considerations	
	No threats were identified in the discussion with the panel	
Acceptability	Research evidence	Variations in acceptability can be anticipated
	No evidence concerning acceptability from patients or surgeons has been found	
	Additional considerations	
	The panel agreed that a bridging repair with composite meshes could be preferred by surgeons despite the well-known risk of recurrence	
Feasibility	Research evidence	Yes
	No evidence regarding feasibility was identified	
	Additional considerations	
	The repair of large defects requires a complex path of preoperative optimisation, not achievable in an emergency [6, 8, 9]	
	Complex abdominal wall reconstruction should preferably be conducted by an expert abdominal wall surgeon to maximise results [63, 64]	
	A subspecialist is often not available in many hospitals, particularly in emergency departments	
	A non-repair strategy frequently necessitates treatments such as negative pressure wound therapy, mesh-mediated fascial traction, and vertical fascial traction, which fall under the expertise of trauma and emergency surgical specialists	
	Hub hospitals offer reliable life support, advanced equipment, and ICU capabilities, making centralisation a possible approach for these cases, according to the panel	

**TABLE 4:** KQ4: Should mesh-based repair vs. no repair be used for the emergency treatment of PVIH in CDC ≥II, stable patients with a defect not amenable to direct closure?

Recommendation		
In stable patients with acutely complicated PVIH with CDC class ≥II wounds and defects not amenable to direct closure, we suggest deferring definitive abdominal wall reconstruction rather than performing immediate reconstructive repair		
Conditional recommendation against repair-Very low certainty of evidence		
Clinical outcomes		
No direct comparative studies between immediate repair and no repair were retrieved. The recommendation relies on indirect evidence of an increased major risk of postoperative complications (CD ≥3b) with immediate repair. See ETD (Table 5) for further explanations		
Evidence for decision framework considerations (nomenclature for judgement on effect is based on GRADE methodology- please see https://gdt.gradepro.org/app/handbook/handbook.html):		
Desirable effects	Research evidence	Judgement
	No direct evidence of desirable effects is available	Varies
	Additional considerations	​
	Source control with early exploration (within 6 h of diagnosis) is the mainstay of treatment for intra-abdominal sepsis caused by gastrointestinal perforations [26, 34]	​
Undesirable effects	Research evidence	Large
	No evidence was retrieved concerning undesirable effects	
	Additional considerations	
	Danish database: In the emergency setting, a tenfold increased risk of complications after retromuscular mesh placement vs. onlay (OR = 10.12, 95% CI = 1.81–56.68, p = 0.008) with increasing rates observed in cases of larger defects and advanced comorbidities [60]	
	AHSQC database: TAR under emergency conditions: High risk of adverse events (wound morbidity - SSO 25.4%, SSI 13.6%, SSOPI 13.6%; reoperation within 30 days: 5.1%; any complication: 37.3%) [61]	
	Concomitant surgery (ventral hernia repair + bowel resection anastomosis) in elective settings yields increased mortality rates, CD 3b and SSI [65]	
Certainty of evidence	Research evidence	Very low
	No direct comparisons between repair and no repair in emergency settings were available in the literature; the evidence used for the recommendation can be considered direct with regard to retromuscular repair under emergency conditions, and indirect with regard to TAR and concomitant procedures, since these were observed in elective settings	
	The certainty of evidence can be considered from low to very low, since it comes from national retrospective registries that are not focused on emergent settings; nevertheless, a large and consistent negative effect was present throughout the articles evaluated when an emergency repair strategy was adopted	
Balance of effects	Research evidence	Likely to favour no repair
	In contaminated or emergent cases, it is safer to perform a separate, staged abdominal wall repair. The high risk of serious complications outweighs the convenience of a single procedure that attempts to fix both the bowel and the abdominal wall at the same time	
Values	Research evidence	Possibly important uncertainty or variability
	No direct evidence has been found in the literature with regard to patient preferences in this setting	
	Additional considerations	
	No variability in patient values and preferences regarding perioperative mortality is anticipated	
	Substantial variation in values and preferences regarding major complications is expected	
	Patient representatives expressed their trust in the surgeon’s choices	
Resources required	Research evidence	Varies in relation to complications and reintervention
	The resources required for simultaneous abdominal wall reconstruction or no repair can vary significantly, as can the costs associated with complications	
Certainty of evidence of required resources	Research evidence	Very low
	The certainty of evidence regarding the required resources is very low. No direct comparisons were made, and only indirect conclusions could be drawn and discussed within the panel	
Equity	Research evidence	Probably no impact
	No evidence regarding equity was identified	
	Additional considerations	
	No threats were identified in the discussion with the panel	
Acceptability	Research evidence	Yes
	No evidence regarding acceptability was identified	
	Additional considerations	
	No threats were identified in the discussion within the panel	
Feasibility	Research evidence	Yes
	No evidence regarding feasibility was identified	
	Additional considerations	
	The panel suggested that there is high variation in the ability to perform the alternatives. The repair of large abdominal wall defects requires techniques and materials that are not frequently available outside experienced centres, namely:	
	• The repair of large, contaminated defects requires a complex path of preoperative optimisation, not achievable in an emergency	
	• Complex abdominal wall reconstruction should preferably be conducted by an expert abdominal wall surgeon to maximise results	
	• A subspecialist is often not available in many hospitals, particularly in emergency departments	
	A non-repair strategy frequently necessitates treatments such as negative pressure wound therapy, mesh-mediated fascial traction, and open abdomen techniques, which fall under the expertise of trauma and emergency surgical specialists	
	• (see EHS guidelines on open abdomen management) [23]	
	• Hub hospitals offer reliable life support, advanced equipment, and ICU capabilities, making centralisation a judicious approach for these cases, according to the panel	

**TABLE 5:** KQ 4a: Should closure of the abdominal cavity vs. open abdomen be used for the emergency treatment of PVIH in CDC ≥II in stable patients with a defect not amenable to direct closure?

Recommendation		
In stable adult patients with acutely complicated PVIH and CDC class ≥II wounds, once adequate source control has been achieved, we suggest immediate closure of the abdominal cavity rather than leaving an open abdomen		
Explanatory note: This can be achieved with skin and sac alone or mediated by mesh in a simple manner after source control has been confirmed. Referral to a hospital with significant expertise in managing complex abdominal wall reconstruction is recommended for further management after the initial life-saving surgery and source control have been achieved		
Conditional recommendation in favour of closure of the abdominal cavity-Very low certainty of evidence- [91]		
Clinical outcomes		
Severe morbidity *(CD ≥3b)*	OR 0.18; 95% CI 0.05–0.70 (1 study- n = 40)	Favours CAC
Reoperation	OR 0.08; 95% CI 0.02–0.37 (1 study-n = 40)	Favours CAC
Evidence for decision framework considerations (nomenclature for judgement on effect is based on GRADE methodology- please see https://gdt.gradepro.org/app/handbook/handbook.html):		
​	Research evidence	Judgement on evidence
Desirable effects	In terms of reoperation and severe complications observed, closure of the abdominal cavity reinforced with resorbable (biological or biosynthetic) mesh is favoured	Likely to favour closure of the abdominal cavity
	Additional considerations	
	Mesh containment with intraperitoneal synthetic mesh as a bridging option is also feasible, but explantation can occur	
	Long-term, fully absorbable meshes in an intraperitoneal position may provide some options	
	The panel endorsed the concept that dissection of the retrorectus or any other plane may increase the complexity of subsequent repair *(burning bridges)*	
Undesirable effects	Research evidence	Varies
	Similar mortality (within 30 days) and fascial closure (within 90 days) have been observed among treatments	
	Additional considerations	
	Open abdomen management carries high complication rates, notably severe enteroatmospheric fistulae, and necessitates ICU treatment. Despite the fact that skin closure prevents visceral exposure, it does not correct fascial retraction or defect enlargement [62]. Therefore, subsequent repair will inherently be a complex abdominal reconstruction	
Certainty of evidence	Research evidence	Very low
	The certainty of evidence was considered to be very low across comparisons of critical outcomes; sensitivity analyses were not possible, as only one retrospective study was available for evaluation	
Balance of effects	Research evidence	Likely to favour closure of the abdominal cavity
	Closure of the abdominal cavity at the time of the procedure prevents visceral exposure to the atmosphere even temporarily, reducing the need for reinterventions and the likelihood of severe complications. The use of a resorbable synthetic or biological mesh allows for better distribution of fascial tension and medialisation of the defect edges, minimising the risk of wound dehiscence and potentially resulting in a definitive, albeit suboptimal, repair. A “planned ventral hernia strategy” may thus be avoided	
Values	Research evidence	Possibly important uncertainty or variability
	No evidence was found regarding patient values	
	Additional considerations	
	No variability in patient values and preferences for perioperative mortality was found	
	Anticipated substantial variation in values and preferences for major complications was found	
	Patient representatives expressed their trust in the surgeon’s choices	
Resources required	Research evidence	Moderate savings
	No evidence on costs or cost-effectiveness was retrieved	
	Additional considerations	
	Closure of the abdominal cavity, whenever feasible and successful, prevents the higher costs associated with adverse events, reoperations, and ICU stays typical of OA.	
	In this scenario, warranted by the likelihood of a definitive repair, the use of a biological or biosynthetic mesh could be justified	
	In each case, both strategies can lead to the management of a complex hernia requiring treatment from expert surgeons in third referral centres	
Certainty of evidence of required resources	Research evidence	Very low
	There is almost no evidence regarding the resources required to implement the technique	
Equity	Research evidence	Probably no impact
	No evidence was found in the literature	
	Additional considerations	
	No concerns about equity are present in the choice of surgical strategy	
Acceptability	Research evidence	Probably yes
	No evidence concerning patient acceptability was retrieved	
	Additional considerations	
	No concerns over acceptability are anticipated	
Feasibility	Research evidence	Probably yes
	No evidence retrieved concerning the feasibility of the two options	
	Additional considerations	
	Closure of the abdominal cavity reinforced with intraperitoneal mesh placement with the parachute technique is a relatively simple technique that shares the main steps of IPOM repair and is feasible for surgeons not specialised in AWR to perform it	
	OA is an effective bailout technique. Nevertheless, the main pitfalls in the adoption of these two approaches are represented by the required experience to choose one over the other and the management of the subsequent treatment pathway	
	OA requires a device and a prolonged stay in the ICU along with strict monitoring	

**TABLE 6:** KQ5: Should retromuscular mesh placement vs. other position mesh placement be used for the emergency repair of acutely complicated PVIH?

Recommendation		
In patients undergoing emergency repair of acutely complicated primary ventral or incisional hernias, we suggest onlay mesh placement rather than retromuscular or other mesh positions, provided a mesh-based repair is chosen		
Conditional recommendation against the sublay mesh position-Very low certainty of evidence- [45, 52, 60]		
Clinical outcomes		
Recurrence	OR 0.62; 95% CI 0.11–3.59 (2 studies-n = 485)	No difference
	OR 2.49; 95% CI 0.20–31.25 (2 studies-n = 619)	No difference
Reoperation	Subgroup analysis:	​
	IH -OR 10.12; 95% CI 1.81–56.68	Favours other position
	PVH -OR 0.76; 95% CI 0.30–1.88	No difference
Evidence for decision framework considerations (nomenclature for judgement on effect is based on GRADE methodology- please see https://gdt.gradepro.org/app/handbook/handbook.html):		
Desirable effects	Research evidence	Judgement on evidence
	There is no significant impact of the mesh plane on short-term recurrence rates in emergency surgery	Varies
	Additional considerations	
	The retromuscular position is associated with improved mesh integration, reduced risk of mesh exposure, and lower recurrence rates in elective surgery. The use of the retromuscular plane can be chosen by experts to treat smaller defects in highly selected cases, even if this method generally requires more dissection and operative time than onlay [27, 66, 67]	
Undesirable effects	Research evidence	Varies
	Retromuscular mesh placement has been suggested to be associated with a significantly increased incidence of reoperation in incisional hernias [60] and a non-statistically significant difference in primary ventral [52]	
	Additional considerations	
	Retromuscular mesh placement is generally considered to be more technically demanding than onlay or intraperitoneal repairs, requiring advanced anatomical dissection and surgical skills [68–70]	
	Onlay placement has been suggested to be associated with an increased risk of SSO and SSI [73]. Emergency priorities (e.g., contamination, bowel resection, or obstruction) may limit the feasibility of an optimal retromuscular repair	
	Using the retromuscular plane may preclude future reconstructive options (“burning bridges”)	
Certainty of evidence	The overall certainty of the evidence was rated as very low for recurrence and low for reoperation	Very low
	Additional considerations	
	Two retrospective studies contributed data to the reoperation analysis, with the pooled results showing no statistically significant difference between the techniques [52, 60]. Notably, Juul et al. [60] reported a tenfold increase in the risk of reoperation due to complications when a retromuscular approach was utilised for emergency incisional hernia repair. However, this effect was mitigated by findings from Fredberg et al. [52] (from the same research group), who focused on primary ventral hernias and observed a comparable risk profile across all repair techniques. This discrepancy likely reflects inherent differences in surgical outcomes based on the type of defect (incisional vs. primary), which explains the lack of significance in the overall meta-analysis. Furthermore, the scarcity of comparative data for other key clinical endpoints limits the robustness of the current evidence base and its utility in guiding definitive surgical decision-making	
Balance of effects	Research evidence	Likely in favour of other mesh positions
	The balance of effects favours other mesh planes (onlay or underlay), based on retrospective studies with a high risk of methodological bias. The panel considered that, despite the advantages offered, retromuscular mesh placement is technically demanding and can result in serious complications, without offering clear advantages in terms of repair stability (recurrence)	
Values	Research evidence	Possibly uncertainty or variability
	No studies were identified that directly assessed patient values or preferences regarding mesh position in emergency ventral hernia repair	
	Additional considerations	
	No important variability is expected with regard to mortality outcomes	
	Important variability in patient preferences with regard to the other outcomes prioritised is expected	
	The patient representative declared that they usually rely on the choices of the surgeon; nevertheless, they would favour a strategy based on an immediate repair to avoid repeated procedures and related risks	
Resources	Research evidence	Moderate costs and savings
	No evidence was retrieved regarding resources or cost-effectiveness of the procedures in an emergent setting	
	Additional Considerations	
	The net impact on healthcare resources was found to be minimal overall	
	Retromuscular dissection requires a longer operative time and technical expertise, while intraperitoneal repair requires more expensive meshes with an anti-adhesive barrier	
	Onlay mesh placement with limited dissection and the use of synthetic, non-reabsorbable mesh can result in reduced operative times and costs	
Certainty of evidence of required resources	Research evidence	Low
	No economic evaluations specific to emergency hernia repair or comparing retrorectus with other mesh placements are available	
Equity	Research evidence	Probably no impact
	No studies were identified that assessed the impact of mesh position on health equity in emergency ventral hernia repair	
	Additional considerations	
	There are no anticipated differences based on socioeconomic status, sex, race/ethnicity, or other population characteristics	
Acceptability	Research evidence	Probably yes
	No studies were identified that directly evaluated the acceptability of retromuscular mesh positioning compared to other mesh positions among patients, surgeons, or other stakeholders in the context of emergency ventral hernia repair	
Feasibility	Research evidence	Varies
	Both included studies demonstrate that retromuscular mesh placement has been performed in emergency ventral hernia repair, providing indirect evidence that the intervention is feasible in selected clinical settings	
	Additional considerations	
	Retrorectus mesh placement is feasible in experienced hands and well-equipped centres. However, in emergency scenarios involving severe contamination, haemodynamic instability, or large defects, feasibility may be reduced	

**TABLE 7:** KQ6: Should synthetic permanent mesh vs. other mesh types be used for the emergency treatment of adult patients with acutely complicated PVIHs?

Recommendation		
In patients undergoing emergency mesh-based repair of acutely complicated primary ventral or incisional hernias, we suggest the use of permanent, macroporous, synthetic mesh rather than other mesh types		
Conditional recommendation in favour of synthetic mesh-The certainty of the evidence ranged from low to very low [44, 50, 51, 71]		
Clinical outcomes		
SSI	OR 0.18; 95% CI 0.05–0.67 (2 studies-n = 173)	Favours synthetic mesh
Mortality	OR 0.75; 95% CI 0.43–1.28 (2 studies- n = 29755)	No difference
Evidence for decision framework considerations (nomenclature for judgement on effect is based on GRADE methodology- please see https://gdt.gradepro.org/app/handbook/handbook.html):		
Desirable effects	Research evidence	Judgement on effect
	Synthetic mesh is associated with a reduced incidence of SSIs. The choice of mesh material does not appear to significantly impact recurrence rates in the short term	Varies
	Additional considerations	
	RCTs produced in elective settings suggest that permanent mesh has a lower recurrence rate in the long term [72].	
Undesirable effects	Research evidence	Trivial
	Current evidence does not demonstrate an increased risk of adverse outcomes with synthetic mesh use	
	Additional considerations	
	Mesh–tissue interaction complications associated with synthetic meshes are documented in the literature (<1–2%) [73, 74]	
Certainty of evidence	The certainty of evidence across the body of literature reviewed was considered to be very low	Very low
Balance of effects	Research evidence	Likely to favour synthetic mesh
	The balance of effects favours synthetic mesh, based on retrospective studies with a high risk of methodological bias. Permanent synthetic mesh offers a theoretical advantage in terms of long-term repair stability in the event of an equivalent rate of SSIs in worst-case scenarios	
Values	Research evidence	Possibly important uncertainty or variability
	No evidence regarding patient values retrieved	
	Additional considerations	
	Patient representatives advocate for the use of materials that offer durable repair and the highest safety profile	
Resources	Research evidence	Moderate savings
	No direct evidence regarding costs was identified in the literature regarding emergency hernia repair	
	Additional Considerations	
	Biologic meshes can cost up to 200 times more than standard synthetic meshes [75]. In a cost analysis from an RCT of elective repair of contaminated hernias, biologic mesh was identified as the primary driver of total 30-day median hospital costs [72]	
Certainty of evidence of required resources	Research evidence	Low
	No evidence retrieved	
Equity	Research evidence	Probably increased
	No direct evidence available for equity	
	Additional considerations	
	The higher costs of biologic and biosynthetic meshes have a clear impact on the equity of treatment	
Acceptability	Research evidence	Varies
	No direct evidence available for acceptability	
	Additional considerations	
	Concerns on acceptability of biological mesh can be highlighted:	
	Patient perspectives: The use of bovine or porcine-derived biological implants represents a major concern from religious (Muslims, hindus, and Sikhs) and cultural perspectives (vegans). Antimesh – MESH out groups oppose all mesh types	
	Surgeons’ perspective: A survey of complex VHR showed that 83.5% of surgeons (France) adopt a suture repair, with the majority of them preferring a biologic mesh for contaminated and dirty cases (consensus 85%) [59]	
	Biosynthetic meshes could be an acceptable option given the promising results published in relation to other types of hernia repair [12], but there is no evidence regarding their use in emergency ventral hernia repair	
Feasibility	Research evidence	Probably yes
	No concerns regarding feasibility have been highlighted	

**TABLE 8:** KQ7: Should a laparoscopic vs. open approach be used for the emergency treatment of stable adult patients with acutely complicated PVIHs?

Recommendation		
In stable adult patients undergoing emergency mesh-based repair of acutely complicated primary ventral or incisional hernias, we suggest a laparoscopic rather than an open surgical approach, when technically feasible		
Explanatory note: Evidence is only available for a laparoscopic repair with intraperitoneal mesh placement		
Conditional recommendation in favour of laparoscopy-the certainty of evidence ranged from moderate to very low [52, 76–82]		
Clinical outcomes		
SSI	OR 0.39; 95% CI 0.33–0.46 (7 studies-n = 41,883)	Favours laparoscopy
Mortality	OR 0.52; 95% CI 0.40–0.68 (5 studies -n = 34,450)	Favours laparoscopy
Reoperation	OR 0.67; 95% CI 0.55–0.81 (5 studies-n = 26,641)	Favours laparoscopy
Recurrence	OR 0.39; 95% CI 0.16–3.48 (2 studies-n = 728)	No difference
Evidence for decision framework considerations (nomenclature for judgement on effect is based on GRADE methodology- please see https://gdt.gradepro.org/app/handbook/handbook.html)		
Desirable effects	Research evidence	Judgement on effect
	There was no evidence of a difference in recurrence rates between laparoscopic and open surgery. The reduced mortality rate recorded for the laparoscopic approach is considered to be biased due to the selection of patients who were better suited to this approach. The laparoscopic approach likely results in little to no difference in reoperation rates after emergency repair of an incisional or ventral hernia. The laparoscopic approach may reduce the risk of SSIs	Small
Undesirable effects	Research evidence	Trivial
	Operative times can vary considerably across studies, reflecting the diverse complexity of emergency hernia presentations	
	Additional considerations	
	A higher rate of missed enterotomies has been observed in the laparoscopic group (one cohort study) [77]	
Certainty of evidence	The certainty of evidence across the body of literature reviewed was considered to be low to very low across comparisons due to limited evidence regarding critical outcomes; sensitivity analyses were therefore not possible	Very low
Balance of effects	Research evidence	Likely to favour intervention
	When feasible, the net balance of effects favoured laparoscopy in terms of patient trauma and early postoperative adverse events	
	Additional considerations	
	No data are available on QoL and postoperative recovery	
	A national survey in the UK revealed a preference for the open approach (62% of surgeons favouring this method)	
	This preference was even more pronounced in complex cases, with a remarkable 97% of cases with bowel perforation identified on radiological examination opting for the open approach [55]	
Values	Research evidence	Possibly important uncertainty or variability
	No evidence retrieved in the available literature	
	Additional considerations	
	The patient representative expressed their appreciation for techniques that reduce trauma and SSIs, and possibly favour recovery when feasible	
Resources	Research evidence	Moderate costs
	Direct evidence comparing the costs of laparoscopic and open emergency ventral hernia repair is lacking in the literature	
	Additional Considerations	
	Where laparoscopic equipment is routinely available, cost is not an issue	
	In settings where laparoscopic equipment is not widely available, cost plays a role and is a limiting factor	
Certainty of evidence of required resources	Research evidence	No included studies
	No direct evidence is available on resource use	
Equity	Research evidence	Probably no impact
	No significant equity concerns have been retrieved in the literature	
	Additional considerations	
	Patients residing in countries with widespread access to laparoscopic surgery are more likely to undergo minimally invasive procedures for acute ventral hernia cases [4, 5]	
	This disparity in access to advanced surgical technology may create inequities in healthcare delivery [83, 84]	
Acceptability	Research evidence	Probably yes
	No evidence regarding acceptability in the literature	
Feasibility	Research evidence	Probably yes
	No evidence regarding feasibility in the literature	
	Additional considerations	
	The fact that laparoscopy is not consistently feasible and the frequently compromised general conditions of the patients limit the adoption of this technique	
	The utilisation of laparoscopic techniques in emergencies has significantly increased in the United States between 2006 and 2016, rising from 7.6% to 17%. Reaching 46% of all emergency ventral hernia repairs by 2019 [79].	

## Discussion

### Implications for Policymakers

A policy of performing emergent mesh repair of acutely complicated PVIH only in cases where the defect can be closed without myofascial release was suggested by this interdisciplinary panel.

Implementation of these guidelines significantly impacts resource allocation. Hospitals should ensure the immediate availability of appropriate mesh types for emergency use.

Utilisation of staged repairs will frequently necessitate prolonged intensive care unit admissions, requiring adequate critical care capacity.

Inter-hospital transfer protocols should be developed with clear policies and funding for the safe and efficient transfer of complex emergency hernia patients from general hospitals to specialised centres. This includes ambulance services, receiving protocols, and communication channels.

Referral to specialised centres for the management of large defects raises the need to identify existing qualified units or develop new ones within the territory. This involves defining criteria for accreditation (e.g., volume, expertise, and outcomes), ensuring equitable access, and potentially developing tiered referral systems along with a clear network for the management of complex abdominal conditions [63].

Furthermore, healthcare systems must meticulously evaluate the cost-effectiveness implications of mesh deployment and extended hospitalisations.

### Implications for Healthcare Professionals

These guidelines, if adopted, could determine a robust shift towards mesh-based repair for defects amenable to closure in emergent settings, challenging the historical reliance on primary suture repair, particularly in clean wounds. For large, non-closable defects, the standard will transition to a staged approach, prioritising temporary abdominal closure (TAC), necessitating surgeon proficiency in these techniques. This constitutes a critical shift in mindset, prioritising physiological stability via a “damage control” approach over immediate definitive reconstruction.

This paradigm shift requires enhanced surgical expertise and a strengthened referral network. Surgeons will require specialised training in emergent mesh-based repairs and TAC. Crucially, the guidelines underscore the importance of recognising when a case exceeds a surgeon’s immediate competency, thereby fostering a culture of referral to specialised centres for the definitive reconstruction of complex defects following initial life-saving intervention. Managing these patients also mandates a multidisciplinary approach, involving collaboration between surgeons, intensivists, and ancillary specialists [64, 85].

Finally, these recommendations carry substantial implications for surgical education and training. Surgical residency and fellowship curricula must integrate these emergency hernia management strategies. Continuing Medical Education (CME) will be paramount for practising surgeons to remain up to date with evolving best practices. Rigorous documentation and systematic audit will be indispensable throughout for monitoring guideline adherence and assessing patient outcomes.

### Implications for Patients

When considering these recommendations in an emergency scenario where patient communication is difficult, their implications shift significantly. Patients, who often lack the capacity for full informed consent due to their critical condition, will heavily rely on surrogate decision-makers, placing a significant burden on families. In these circumstances, the necessity of the recommended intervention, driven by guidelines aimed at optimising survival and minimising severe complications, will take precedence over individual preferences. Patients will have limited ability to process complex information, highlighting the importance of post-operative education to explain procedures. They will be highly dependent on the surgical team’s judgement and trust in pre-defined, evidence-informed protocols. Crucially, they must be prepared for a potentially longer and more complex recovery journey involving multiple stages and possibly transfers to specialised facilities. This underscores the ethical imperative for meticulous documentation of urgent decisions and transparent communication with available family members.

### Implications for Researchers

Despite the extensive work of research performed by the guidelines development group, several issues related to PVIH treatment under emergency conditions were encountered, particularly with regard to methodological bias concerning the selection of patients, which precludes definitive conclusions. In particular, biases that were already highlighted in elective settings were found to be more prevalent in studies on the management of emergency hernias. Several articles included mixed cohorts in terms of defect type (inguinal vs. ventral; primary vs. incisional), patient risk profile, type of mesh used and contamination grade. Evidence regarding the robotic approach to emergency abdominal wall hernias is limited to some non-comparative studies, and this could represent a possible future area of research [86].

Future research should target:

- Mesh repair vs. suture repair
- Different mesh positions and materials
- The management of defects amenable to closure
- Quality of life after emergency PVIH repair
- Concomitant repair vs. staged repair in defects not amenable to closure in a clean scenario
- Synthetic permanent mesh vs. biosynthetic or biological mesh in an emergent contaminated scenario
- Open vs. minimally invasive surgery in the management of emergent PVIHs.

We recommend conducting matched, multicentre studies to ensure adequate statistical power and to obtain precise effect estimates.

### Barriers and Facilitators

Surgeon expertise and training gaps, along with clinical routine, probably represent the primary obstacles [87]. In particular, the lack of interest in innovations in advanced abdominal wall reconstruction techniques and temporary abdominal closure among general surgeons, especially in smaller or non-specialised hospitals, is considered the main issue. The shift towards mesh-based repair in emergencies and staged approaches requires experience, encompassing both abdominal wall surgery and damage control strategies, which many surgeons may not possess.

Moreover, implementing these guidelines demands specific resources that may not be universally available. These include access to diverse mesh types in emergency stock, sufficient ICU bed capacity for prolonged post-operative care in staged approaches, and specialised equipment.

Financial and reimbursement challenges may also be an issue, since the recommended approaches can be more expensive in the short term due to the cost of mesh, longer hospital stays (especially in the ICU), and the need for multiple procedures in staged repairs. Current reimbursement models may not adequately cover these complex, multi-stage treatments, potentially creating disincentives for hospitals and surgeons.

Referral system limitations can represent a relevant barrier: the emphasis on referring complex cases to specialised centres relies on a robust and efficient inter-hospital transfer system. Barriers could include a lack of established referral pathways, limited availability of specialised centres, or logistical difficulties in transferring critically ill patients over long distances.

Finally, communication and shared decision-making with patients or their surrogates in emergency settings is already challenging. Explaining complex, multi-stage plans with nuanced benefits and risks can be a significant hurdle.

The EHS has courses and fellowships across Europe designed to fill the knowledge gap on this topic. These are held by experts in abdominal wall and trauma surgery. These will help by increasing the availability of surgeons with sufficient skills to manage acutely complicated abdominal wall defects. The financial accessibility of this type of training, facilitated by policymaker support, is essential for building the necessary expertise. While extensive patient-centred decision aids are difficult to implement in an acute setting, the underlying principles of clear, concise communication become crucial when engaging with surrogate decision-makers, helping them to quickly understand the urgent situation and the rationale behind critical treatment choices. The EHS Publication Wing has developed materials aimed at clarifying the aspects of the intervention discussed in this document, for both surgeons and patients.

### Monitoring

Use of the guidelines will be monitored by the Executive Board of the EHS through an online questionnaire sent to members within 3 years of publication from the Social Media Wing of the Society. Additionally, use of the guidelines will be monitored through direct citations of each article and dedicated metrics from the Journal of Abdominal Wall Surgery. Any feedback received from target users in the form of email communication, publications, and social media engagement will be documented to inform future versions of the present document. We advise monitoring the implementation of these interventions at all respective institutions and establishing clinical outcomes among surgeons at all institutions for the purpose of quality improvement.

### Validity Period

A scoping search of ClinicalTrials.gov (conducted on 30 June 2025) for trials on emergency PVIH management returned no currently active studies. The most recent publication in this area is Crepaz et al.'s work in Updates in Surgery this year [54]. While a scoping review by Quiroga in Hernia [7] indicates a rising trend in publications on this topic, with an average of five articles per year, not all of these are comparative studies. Given the absence of ongoing trials and the nature of recent publications, it is unlikely that new trials with a substantial impact on the current evidence base will be completed within the next 5 years. Therefore, the recommendations of the present guidelines for emergency ventral hernia management are valid until June 2030.

### Updates

We plan to update these guidelines in 2031, unless substantial new evidence becomes available.

## Conclusion

These guidelines provide recommendations on the management of acutely complicated PVIH based on the best available evidence; they were developed by an interdisciplinary international panel of stakeholders using a structured, trustworthy methodology.

## Footnotes

1
https://gdt.gradepro.org/app/

## Author Disclaimer

These clinical practice guidelines have been developed under the auspices of the European Hernia Society (EHS). They are intended to be used primarily by health professionals (e.g., surgeons, anaesthetists, and physicians) to assist them in making informed clinical decisions on therapeutic management. They are also intended to inform the individual practice of allied health professionals (e.g., surgical nurses, dieticians, physical rehabilitation therapists, and psychologists) [88, 89], inform the strategic planning and resource management of healthcare authorities (e.g., regional and national authorities, healthcare institutions, and hospital administration authorities); and inform patients who wish to gain an understanding of their condition and its management. The use of the recommendations contained herein must be informed by supporting evidence accompanying each recommendation, along with the research evidence that may not have been published by the time the present document was written. Users must thus base their actions on newly published evidence at any given point in time. The information in the guidelines should not be relied upon as being complete or accurate, nor should it be considered to be inclusive of all proper treatments or methods of care or as a statement of the standard of care. With the rapid development of scientific knowledge, new evidence may emerge between the time the guidelines are developed and when they are published or read [90]. The guidelines are not continually updated and may not reflect the most recent evidence. The guidelines address only the topics specifically identified within them and are not applicable to other interventions, diseases, or disease stages. These guidelines do not mandate any particular course of medical care. Furthermore, the guidelines are not intended to replace the independent professional judgement of the treating provider, as the guidelines do not necessarily account for individual variation between patients. Even if the evidence regarding a topic suggests a specific diagnostic and/or treatment approach, users and especially health professionals may need to decide against the suggested or recommended action in view of circumstances relating to patient values, preferences, co-morbidities, and disease characteristics; available human, financial and material resources; and healthcare infrastructures. EHS provides these guidelines on an “as is” basis and makes no warranty, either express or implied, regarding the guidelines. Patients and other stakeholders—particularly regulatory agencies or the courts—should not view the recommendations in these guidelines as unconditional, because no guideline can account for the unique features of individual patients and their clinical circumstances; these guidelines are designed to aid, not replace, good clinical judgement.
