# Supplementary material for: EHS Guidelines on the Management of Primary Ventral and Incisional Hernias Under Emergency Conditions
Source: J Abdom Wall Surg. 2026 Mar 11;5:16228. doi: 10.3389/jaws.2026.16228 (PMC13044802; doi:10.3389/jaws.2026.16228)
Supplement: Supplementary file 1 [file Supplementaryfile6.docx]

**Supplementary file 6- Study included for each KQs**

For KQ1, our analysis incorporated one RCT(1), one prospective study (2), and eight retrospective case-control studies (3, 4, 5, 6, 7, 8, 9, 10).

KQ2 was informed by four retrospective case-control studies (4, 11, 12, 13).

KQ3 included one retrospective four-arm case-control study(14)

No studies were identified for KQ4, necessitating the inclusion of one prospective case-control study for KQ4a (15).

For KQ5, three retrospective case-control studies were included (3, 10, 16).

KQ6 was supported by one prospective and three retrospective case-control studies (2, 8, 9, 17).

KQ7 drew evidence from eight retrospective case-control studies(10, 18, 19, 20, 21, 22, 23, 24)

References

1. Abdel-Baki NA, Bessa SS, Abdel-Razek AH. Comparison of prosthetic mesh repair and tissue repair in the emergency management of incarcerated para-umbilical hernia: a prospective randomized study. Hernia. 2007;11(2):163-7.10.1007/s10029-007-0189-4

2. Proctor VK, O'Connor OM, Burns FA, Green S, Sayers AE, Hawkins DJ, et al. Management of Acutely Symptomatic Hernia (MASH) study. Br J Surg. 2022;109(8):754-62.10.1093/bjs/znac107

3. Dissanayake B, Burstow MJ, Yuide PJ, Gundara JS, Chua TC. Early outcomes of emergency ventral hernia repair in a cohort of poorly optimized patients. ANZ journal of surgery. 2020;90(7-8):1447-53.<https://dx.doi.org/10.1111/ans.16020>

4. Emile SH, Elgendy H, Sakr A, Gado WA, Abdelmawla AA, Abdelnaby M, et al. Outcomes following repair of incarcerated and strangulated ventral hernias with or without synthetic mesh. World journal of emergency surgery : WJES. 2017;12:31.<https://dx.doi.org/10.1186/s13017-017-0143-4>

5. La Mura F, Cirocchi R, Farinella E, Morelli U, Napolitano V, Cattorini L, et al. Emergency treatment of complicated incisional hernias: a case study. Annals of surgical innovation and research. 2009;3:15.<https://dx.doi.org/10.1186/1750-1164-3-15>

6. Nieuwenhuizen J, Van Ramshorst GH, Ten Brinke JG, De Wit T, Van Der Harst E, Hop WCJ, et al. The use of mesh in acute hernia: Frequency and outcome in 99 cases. Hernia. 2011;15(3):297-300.10.1007/s10029-010-0779-4

7. Odom SR, Gupta A, Talmor D, Novack V, Sagy I, Evenson AR. Emergency hernia repair in cirrhotic patients with ascites. The journal of trauma and acute care surgery. 2013;75(3):404-9.10.1097/ta.0b013e31829e2313

8. Ricard CA, Aalberg JJ, Bawazeer MA, Johnson BP, Hojman HM, Kim WC, et al. Readmissions after emergent incisional ventral hernia repair: a retrospective review of the nationwide readmissions database. Updates in surgery. 2023;75(7):1979-89.<https://dx.doi.org/10.1007/s13304-023-01469-9>

9. Whittaker R, Lewis Z, Plymale MA, Nisiewicz M, Ebunoluwa A, Davenport DL, et al. Emergent and urgent ventral hernia repair: comparing recurrence rates amongst procedures utilizing mesh versus no mesh. Surgical endoscopy. 2022;36(10):7731-7.<https://dx.doi.org/10.1007/s00464-022-09101-4>

10. Fredberg J, Oma E, Helgstrand F, Qvist N, Friis-Andersen H, Jorgensen LN. Emergency umbilical and epigastric hernia repair: nationwide registry-based study of long-term recurrence, mesh-related, and other complications. Surg Endosc. 2025;39(7):4253-65.10.1007/s00464-025-11792-4

11. Haskins IN, Amdur RL, Lin PP, Vaziri K. The Use of Mesh in Emergent Ventral Hernia Repair: Effects on Early Patient Morbidity and Mortality. Journal of gastrointestinal surgery : official journal of the Society for Surgery of the Alimentary Tract. 2016;20(11):1899-903.<https://dx.doi.org/10.1007/s11605-016-3207-y>

12. Surek A, Gemici E, Ferahman S, Karli M, Bozkurt MA, Dural AC, et al. Emergency surgery of the abdominal wall hernias: risk factors that increase morbidity and mortality—a single-center experience. Hernia. 2021;25(3):679-88.10.1007/s10029-020-02293-5

13. Tomaoglu K, Okmen H. Prosthetic mesh hernioplasty versus primary repair in incarcerated and strangulated groin and abdominal wall hernias with or without organ resection. Retrospective study. Langenbeck's Archives of Surgery. 2021;406(5):1651-7.10.1007/s00423-021-02145-5

14. Coccolini F, Paratore F, Tartaglia D, Cremonini C, Zocco G, Cobuccio L, et al. Biological prosthesis, platelet enriched plasma and bone marrow stem cells in complicated incisional hernia reconstruction in emergency surgery: a prospective case control study. Emergency Care Journal. 2023;19(1).10.4081/ecj.2023.11050

15. de Vries FEE, Claessen JJM, Atema JJ, van Ruler O, Boermeester MA, Group C-US. Immediate Closure of Abdominal Cavity with Biologic Mesh versus Temporary Abdominal Closure of Open Abdomen in Non-Trauma Emergency Patients (CLOSE-UP Study). Surg Infect (Larchmt). 2020;21(8):694-703.10.1089/sur.2019.289

16. Juul N, Henriksen NA, Jensen KK. Increased risk of postoperative complications with retromuscular mesh placement in emergency incisional hernia repair: A nationwide register-based cohort study. Scandinavian journal of surgery : SJS : official organ for the Finnish Surgical Society and the Scandinavian Surgical Society. 2021;110(2):193-8.<https://dx.doi.org/10.1177/1457496920966237>

17. Loftus TJ, Go KL, Jordan JR, Croft CA, Smith RS, Moore FA, et al. Computed tomography evidence of fluid in the hernia sac predicts surgical site infection following mesh repair of acutely incarcerated ventral and groin hernias. The journal of trauma and acute care surgery. 2017;83(1):170-4.<https://dx.doi.org/10.1097/TA.0000000000001503>

18. Alwadaani HA, Memon AQ. Outcome of the incarcerated abdominal wall hernias managed by open and laparoscopic approaches. Pakistan Journal of Medical Sciences. 2024;40(5):946-50.10.12669/pjms.40.5.8899

19. Azin A, Hirpara D, Jackson T, Okrainec A, Elnahas A, Chadi SA, et al. Emergency laparoscopic and open repair of incarcerated ventral hernias: a multi-institutional comparative analysis with coarsened exact matching. Surgical endoscopy. 2019;33(9):2812-20.<https://dx.doi.org/10.1007/s00464-018-6573-6>

20. Jacob R, Guy SB, Kamila L, Idan C, Shlomi R, Youri M. Comparison of emergent laparoscopic and open repair of acutely incarcerated and strangulated hernias—short- and long-term results. Surgical Endoscopy. 2023;37(3):2154-62.10.1007/s00464-022-09743-4

21. Kao AM, Huntington CR, Otero J, Prasad T, Augenstein VA, Lincourt AE, et al. Emergent Laparoscopic Ventral Hernia Repairs. The Journal of surgical research. 2018;232:497-502.<https://dx.doi.org/10.1016/j.jss.2018.07.034>

22. Pechman DM, Cao L, Fong C, Thodiyil P, Surick B. Laparoscopic versus open emergent ventral hernia repair: utilization and outcomes analysis using the ACSNSQIP database. Surgical endoscopy. 2018;32(12):4999-5005.<https://dx.doi.org/10.1007/s00464-018-6312-z>

23. Proaño-Zamudio JA, Gebran A, Argandykov D, Paranjape CN, Maroney SJ, Onyewadume L, et al. Complicated Abdominal Wall Hernias in the Elderly: Time Is Life and Comorbidities Matter. American Surgeon. 2023;89(6):2529-36.10.1177/00031348221101577

24. Kaoutzanis C, Leichtle SW, Mouawad NJ, Welch KB, Lampman RM, Cleary RK. Postoperative surgical site infections after ventral/incisional hernia repair: a comparison of open and laparoscopic outcomes. Surgical endoscopy. 2013;27(6):2221-30.<https://dx.doi.org/10.1007/s00464-012-2743-0>
