# Supplementary material for: EHS Guidelines on the Management of Primary Ventral and Incisional Hernias Under Emergency Conditions
Source: J Abdom Wall Surg. 2026 Mar 11;5:16228. doi: 10.3389/jaws.2026.16228 (PMC13044802; doi:10.3389/jaws.2026.16228)
Supplement: Supplementary file 3 [file Supplementaryfile10.docx]

**Supplementary file 10**

| **Summary of findings table KQ2** | | | | | | |
| --- | --- | --- | --- | --- | --- | --- |
| **Mesh Based Repair compared to tissue repair in emergency primary ventral and incisional hernia repair for defects amenable to closure in CDC ≥2 wound class** | | | | | | |
| Outcomes | **Anticipated absolute effects^*^** (95% CI) | | Relative effect (95% CI) | № of participants (studies) | Certainty of the evidence (GRADE) | Comments |
|  | **Risk with Primary Fascial Closure** | **Risk with Mesh Based Repair** |  |  |  |  |
| Mortality | 42 per 1.000 | **28 per 1.000** (8 to 98) | **OR 0.67** (0.18 to 2.49) | 426 (1 non-randomised study) | ⨁◯◯◯ Very low^a,b^ | Mesh Based Repair may reduce/have little to no effect on mortality but the evidence is very uncertain. |
| SSI | 146 per 1.000 | **167 per 1.000** (137 to 201) | **OR 1.17** (0.93 to 1.47) | 2820 (3 non-randomised studies) | ⨁⨁◯◯ Low^a,c^ | Mesh Based Repair may not increase SSI. |
| Recurrence | 127 per 1.000 | **34 per 1.000** (13 to 82) | **OR 0.24** (0.09 to 0.61) | 371 (2 non-randomised studies) | ⨁◯◯◯ Very low^c,d^ | Mesh Based Repair may reduce/have little to no effect on recurrence but the evidence is very uncertain. |
| ***The risk in the intervention group** (and its 95% confidence interval) is based on the assumed risk in the comparison group and the **relative effect** of the intervention (and its 95% CI).  **CI:** confidence interval; **OR:** odds ratio | | | | | | |

#### Explanations

a. more than 50% of papers are at least at moderate risk of bias

b. confidence interval crossing decision threshold(s)

c. low numbers of patients enrolled and events

d. more than 50% of papers are at serious risk of bias

mortality

SSI

recurrence
