# Supplementary material for: EHS Guidelines on the Management of Primary Ventral and Incisional Hernias Under Emergency Conditions
Source: J Abdom Wall Surg. 2026 Mar 11;5:16228. doi: 10.3389/jaws.2026.16228 (PMC13044802; doi:10.3389/jaws.2026.16228)
Supplement: Supplementary file 4 [file Supplementaryfile13.docx]

**Supplementary file 13**

| **Summary of findings KQ5** | | | | | | |
| --- | --- | --- | --- | --- | --- | --- |
| **Retrorectus/retromuscular compared to other mesh positions in the Mesh Based Repair of emergency primary ventral and incisional hernia** | | | | | | |
|  | | | | | | |
| Outcomes | **Anticipated absolute effects^*^** (95% CI) | | Relative effect (95% CI) | № of participants (studies) | Certainty of the evidence (GRADE) | Comments |
|  | **Risk with other mesh positions** | **Risk with retrorectus/retromuscular** |  |  |  |  |
| Recurrence | 16 per 1000 | **10 per 1000** (2 to 57) | **OR 0.62** (0.11 to 3.59) | 485 (2 non-randomised studies) | ⨁◯◯◯ Very low^a,b,c^ | Retrorectus/retromuscular may increase/have little to no effect on recurrence but the evidence is very uncertain. |
| Reoperation overall | 0 per 1000 | **0 per 1000** (0 to 0) | **OR 2.49** (0.20 to 31.25) | 619  (2 non-randomised studies) | ⨁◯◯◯ Very low^a,c,e^ | Retrorectus/retromuscular may increase/have little to no effect on reoperation but the evidence is very uncertain. |
| *Reoperation IH* | *58 per 1000* | ***383 per 1000*** *(100 to 777)* | ***OR 10.12*** *(1.81 to 56.68)* | *168 (1 non-randomised study)* | *⨁⨁◯◯ Low^a,c,d^* | *Retrorectus/retromuscular may result in an increase in reoperation.* |
| *Reoperation PVH* | *79 per 1000* | ***61 per 1000*** *(25 to 139)* | ***OR 0.76*** *(0.30 to 1.88)* | *451 (1 non-randomised study)* | *⨁◯◯◯ Very low^c,d^* | *Retrorectus/retromuscular may increase/have little to no effect on reoperation but the evidence is very uncertain.* |
| ***The risk in the intervention group** (and its 95% confidence interval) is based on the assumed risk in the comparison group and the **relative effect** of the intervention (and its 95% CI).  **CI:** confidence interval; **OR:** odds ratio | | | | | | |

#### Explanations

a. More than 60% of papers are at serious/very serious risk of bias

b. low heterogeneity across studies was retrieved (I2 0%)

c. Very few events, very wide CI

d. Only one study was available, precluding assessment of consistency across studies

e. high heterogeneity detected I2=85%

recurrence

reoperation
