# Supplementary material for: EHS Guidelines on the Management of Primary Ventral and Incisional Hernias Under Emergency Conditions
Source: J Abdom Wall Surg. 2026 Mar 11;5:16228. doi: 10.3389/jaws.2026.16228 (PMC13044802; doi:10.3389/jaws.2026.16228)
Supplement: Supplementary file 5 [file Supplementaryfile1.docx]

Supplementary file 1

| 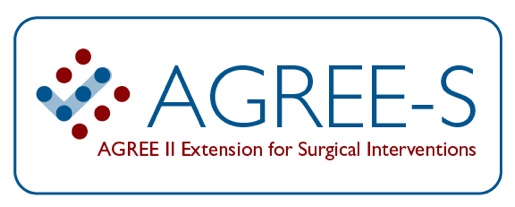 | AGREE-S Reporting Checklist |
| --- | --- |

Title of guideline: *EHS Guideline on the management of Ventral Hernia under emergency conditions*

Name of first author: *Cesare Stabilini*

Sponsoring organization (if applicable): *not applicable*

| **Section** | **Checklist item** | **✔︎** | **Reported in:**  **(page # or manuscript section)** |
| --- | --- | --- | --- |
| Protocol | 1. The guideline has been developed according to a protocol and the link to the protocol is provided. | x | Material and methods |
| Objective | 1. The overall objective(s) of the guideline is (are) specifically described. | x | introduction |
| Health question(s) | 1. The health question(s) covered by the guideline [patient, interventions/procedures, outcomes] are specifically described. | x | Material and methods |
| Methodological support | 1. The guideline reports on whether it was supported by a guideline development committee, including a guideline methodologist. | x | introduction |
| Stakeholder involvement | 1. Representation of professional groups and patients included in the guideline development group is reported. | x | Material and methods |
| Target users | 1. The target users of the guideline are specifically described. | x | Material and methods |
| Systematic review | 1. The methods that were used to search for evidence are clearly described. | x | Material and methods +suppl file 4 |
| Selection criteria | 1. The criteria for selecting the evidence are clearly described. | x | Material and methods +suppl file 4 |
| Strengths/limitations of evidence | 1. The strengths and limitations of the body of evidence are clearly described. | x | Results +suppl file 8-15 |
| Patient/public input | 1. The views and preferences of the target population (patients, public, etc.) are reported. | x | tables 1-7 |
|  | | | |
| **Section** | **Checklist item** | **✔︎** | **Reported in:**  **(e.g. page # or manuscript section)** |
| Formulation of recommendations | 1. The methods for formulating the recommendations are clearly described. | x | Material and methods |
| Link between evidence and recommendations | 1. The health benefits, side effects, and risks have been considered in formulating the recommendations. | x | Results+tables 1-7 |
| Link between evidence and recommendations | 1. The link between the recommendations and the supporting evidence is explicitly reported. | x | Results+tables 1-7+supplementary files 9-15 |
| Clarity of recommendations | 1. The recommendations are specific and unambiguous. | x | Tables 1-7 |
| Alternative options | 1. The different options for management of the condition or health issue are clearly presented. | x | Tables 1-7 |
| Identification of recommendations | 1. Key recommendations are easily identifiable. | x | Tables 1-7 |
| Resource considerations | 1. The potential resource implications of applying the recommendations have been considered. | x | Table 1-7 |
| Practice variability | 1. The guideline discusses potential variability in surgical expertise of those performing the interventions/procedures. | x | Discussion Tables 1-7 |
| Role of funder | 1. The role of the funding body is described. | x | discussion |
| Conflicts of interest | 1. Competing interests of guideline development group members are reported in detail. | x | discussion |
| Facilitators and barriers | 1. The guideline describes facilitators and barriers to its application. | x | discussion |
| Update | 1. A procedure for updating the guideline is provided. | x | discussion |
| Implementation | 1. The guideline provides advice and/or tools on how the recommendations can be put into practice. | x | figure 1+ table 1-7 |
| Monitoring | 1. The guideline presents monitoring and/or auditing criteria. | x | discussion |

Copyright: The GAP Consortium
