# Supplementary material for: EHS Guidelines on the Management of Primary Ventral and Incisional Hernias Under Emergency Conditions
Source: J Abdom Wall Surg. 2026 Mar 11;5:16228. doi: 10.3389/jaws.2026.16228 (PMC13044802; doi:10.3389/jaws.2026.16228)
Supplement: Supplementary file 7 [file Supplementaryfile11.docx]

**Supplementary file 11**

| **Summary of findings KQ3** | | | | | | |
| --- | --- | --- | --- | --- | --- | --- |
| **Mesh based repair compared to No repair in emergency primary ventral and incisional hernia repair for defects not amenable to closure in CDC 1 wound class** | | | | | | |
| Outcomes | **Anticipated absolute effects^*^** (95% CI) | | Relative effect (95% CI) | № of participants (studies) | Certainty of the evidence (GRADE) | Comments |
|  | **Risk with No repair** | **Risk with Repair** |  |  |  |  |
| Mortality | not pooled | not pooled | not pooled | 40 (1 non-randomised study) | ⨁◯◯◯ Very low^a,b^ |  |
| Recurrence | 600 per 1.000 | **95 per 1.000** (15 to 387) | **OR 0.07** (0.01 to 0.42) | 40 (1 non-randomised study) | ⨁◯◯◯ Very low^a,b^ | Repair may reduce/have little to no effect on recurrence but the evidence is very uncertain. |
| Reoperation | 100 per 1.000 | **11 per 1.000** (0 to 235) | **OR 0.10** (0.00 to 2.77) | 40 (1 non-randomised study) | ⨁◯◯◯ Very low^a,b^ | Repair may have little to no effect on reoperation but the evidence is very uncertain. |
| Morbidity (Clavien Dindo>3b) | 200 per 1.000 | **15 per 1.000** (0 to 242) | **OR 0.06** (0.00 to 1.28) | 40 (1 non-randomised study) | ⨁◯◯◯ Very low^a,b^ | Repair may have little to no effect on morbidity but the evidence is very uncertain. |
| ***The risk in the intervention group** (and its 95% confidence interval) is based on the assumed risk in the comparison group and the **relative effect** of the intervention (and its 95% CI).  **CI:** confidence interval; **OR:** odds ratio | | | | | | |

#### Explanations

a. more than 50% of the studies is at moderate or serious risk of bias

b. very few events and low sample size

**recurrence**
