# Supplementary material for: EHS Guidelines on the Management of Primary Ventral and Incisional Hernias Under Emergency Conditions
Source: J Abdom Wall Surg. 2026 Mar 11;5:16228. doi: 10.3389/jaws.2026.16228 (PMC13044802; doi:10.3389/jaws.2026.16228)

**Supplementary file 9**

| **Summary of findings table KQ1** | | | | | | |
| --- | --- | --- | --- | --- | --- | --- |
| **Mesh Based Repair compared to Primary Fascial Closure in emergency primary ventral and incisional hernia repair for defects amenable to closure in CDC 1 wound class** | | | | | | |
|  | | | | | | |
| Outcomes | **Anticipated absolute effects^*^** (95% CI) | | Relative effect (95% CI) | № of participants (studies) | Certainty of the evidence (GRADE) | Comments |
|  | **Risk with Primary Fascial Closure** | **Risk with Mesh Based Repair** |  |  |  |  |
| MORBIDITY (CLAVIEN DINDO ≥ 3B) | 34 per 1.000 | **82 per 1.000** (206 to 30) | **OR 2.55**  (0.88 to 7.39) | 708 (2 non-randomised study) | ⨁⨁◯◯ Low^a,b^ | Mesh Based Repair may result in little to no difference in MORBIDITY (CLAVIEN DINDO >3B) |
| MORTALITY | 25 per 1.000 | **10 per 1.000** (8 to 11) | **OR 0.38** (0.33 to 0.45) | 43861 (2 non-randomised studies) | ⨁⨁⨁◯ Moderate^c^ | Mesh Based Repair likely reduces MORTALITY slightly. |
| SSI | 92 per 1.000 | **40 per 1.000** (15 to 105) | **OR 0.41** (0.15 to 1.15) | 399 (4 non-randomised studies) | ⨁⨁◯◯ Low^a,d^ | Mesh Based Repair may result in a slight reduction in SSI. |
| RECURRENCE | 6 per 1.000 | **3 per 1.000** (2 to 4) | **OR 0.47** (0.31 to 0.72) | 45037 (8 non-randomised studies) | ⨁⨁◯◯ Low^a,d^ | Mesh Based Repair may result in a slight reduction in RECURRENCE. |
| REOPERATION | 38 per 1.000 | **52 per 1.000** (25 to 104) | **OR 1.41** (0.67 to 2.99) | 729 (2 non-randomised studies) | ⨁⨁◯◯ Low^a,d^ | Mesh Based Repair may result in little to no difference in REOPERATION. |
| ***The risk in the intervention group** (and its 95% confidence interval) is based on the assumed risk in the comparison group and the **relative effect** of the intervention (and its 95% CI).  **CI:** confidence interval; **OR:** odds ratio | | | | | | |

#### Explanations

a. more than 50% of the risk of bias of the entire group of studies is at moderate to serious ROB

b. imprecision due to small number of events and participants

c. Overall Risk of bias resulted Moderate at ROB2 assessment

d. imprecision due to small number of events and wide CI

**morbidity**


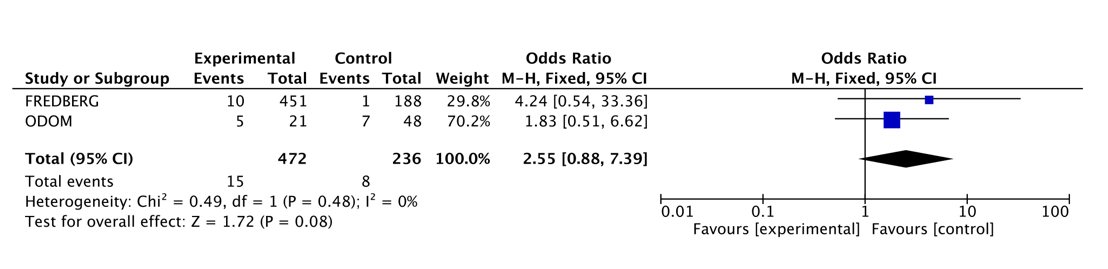
mortality
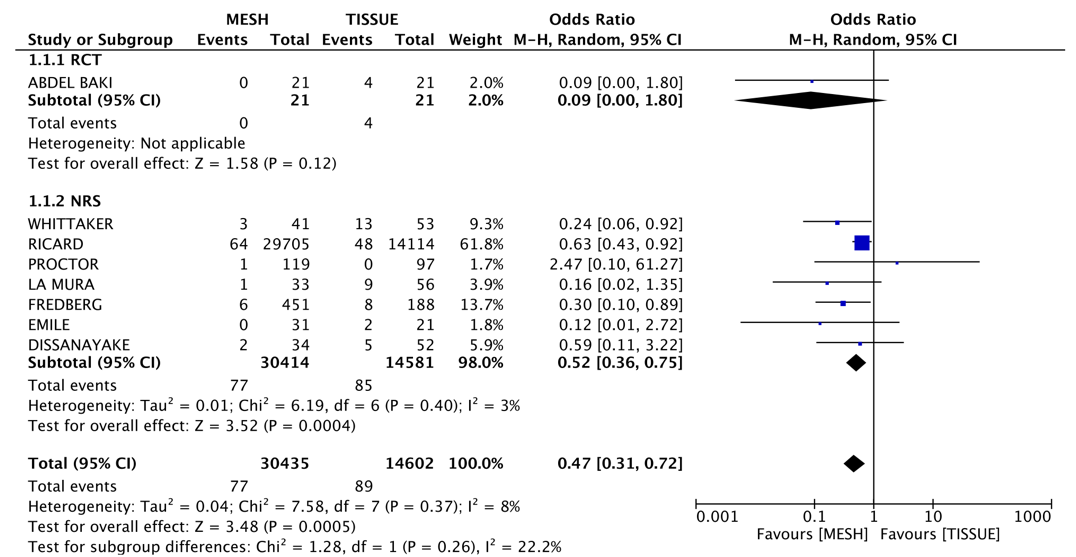


SSI


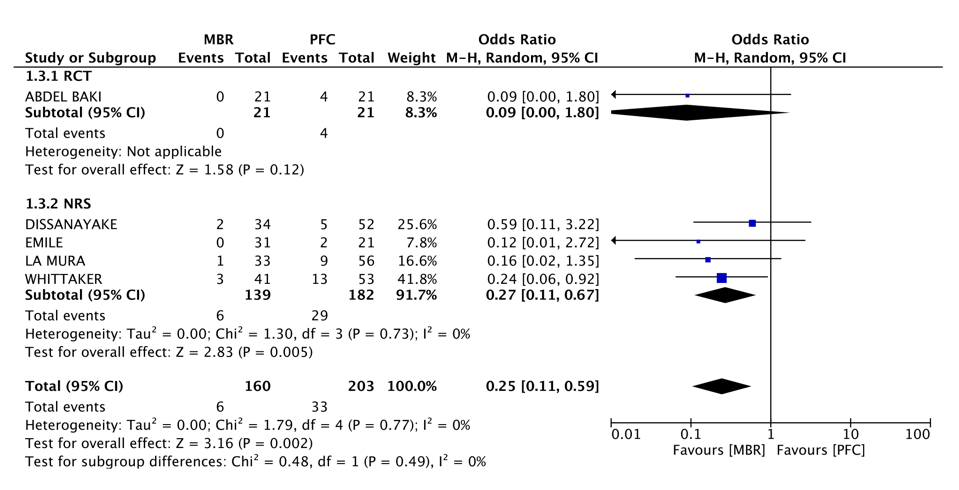


Recurrence


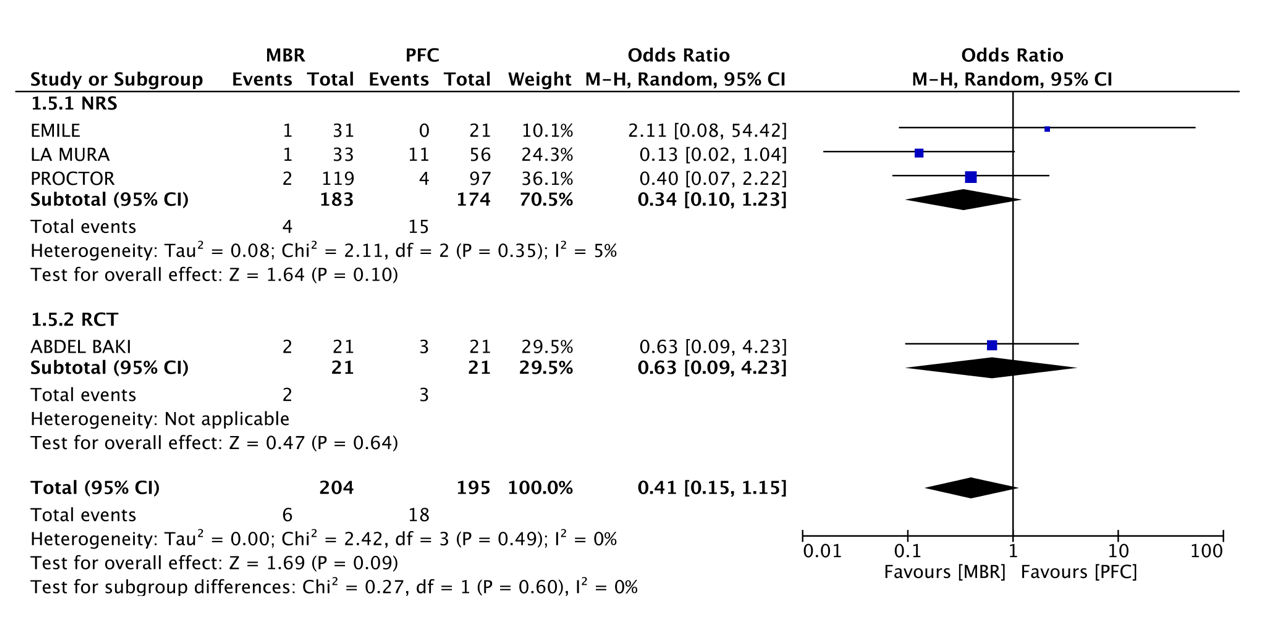


Reoperation


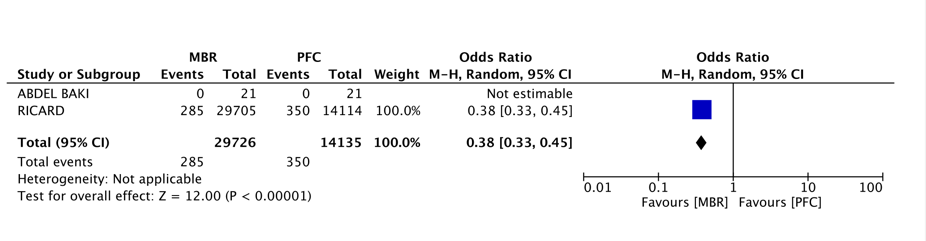

Supplement: Supplementary file 8 [file Supplementaryfile9.docx]
