# Supplementary material for: EHS Guidelines on the Management of Primary Ventral and Incisional Hernias Under Emergency Conditions
Source: J Abdom Wall Surg. 2026 Mar 11;5:16228. doi: 10.3389/jaws.2026.16228 (PMC13044802; doi:10.3389/jaws.2026.16228)
Supplement: Supplementary file 9 [file Supplementaryfile7.docx]

**Supplementary file 7- PRISMA 2020 Flow diagram**

**Identification of studies via databases**

Records removed *before screening*:

Duplicate records removed (n =98 )

Records marked as ineligible by automation tools (n = 0)

Records removed for other reasons (n = 0)

Records identified from*:

Databases (n = 2000)

**Identification**

Records screened

(n = 1902)

Records excluded**

(n = 1726 )

Reports sought for retrieval

(n =176)

Reports not retrieved

(n =5 )

**Screening**

Reports assessed for eligibility

(n = 171)

Reports excluded: (n=147)

Studies included in review

(n = 24 )

Reports of included studies

(n = 24)

**Included**

*Consider, if feasible to do so, reporting the number of records identified from each database or register searched (rather than the total number across all databases/registers).

**If automation tools were used, indicate how many records were excluded by a human and how many were excluded by automation tools.

Source: Page MJ, et al. BMJ 2021;372:n71. doi: 10.1136/bmj.n71.

This work is licensed under CC BY 4.0. To view a copy of this license, visit <https://creativecommons.org/licenses/by/4.0/>
