# Supplementary material for: EHS Guidelines on the Management of Primary Ventral and Incisional Hernias Under Emergency Conditions
Source: J Abdom Wall Surg. 2026 Mar 11;5:16228. doi: 10.3389/jaws.2026.16228 (PMC13044802; doi:10.3389/jaws.2026.16228)
Supplement: Supplementary file 10 [file Supplementaryfile2.docx]

**Supplementary file 2**

**Guideline Development Group –Composition and roles**

| **Emergency GDG (22 members)** | | |  |  |
| --- | --- | --- | --- | --- |
| **Steering Comittee** | C. Stabilini  A. Theodorou  E. Deerenberg  M. Pawlak | General and Hernia Surgeon  General and Hernia Surgeon  General and Hernia Surgeon  General and Hernia Surgeon | ITA  GRE  NL  POL/GBR | MALE  MALE  FEMALE  MALE |
| **Evidence Review Team – Clinical** | A.C. Quiroga Centeno  K. Gilmore  S.Capoccia Giovannini | General Surgeon  General Surgeon  General and Hernia Surgeon | COL  GBR  ITA | FEMALE  FEMALE  FEMALE |
| **Evidence Review Team – ETD** | F. Pecchini  E. Schembari  *(S.Capoccia Giovannini)* | General Surgeon  General Surgeon  *General and Hernia Surgeon* | ITA  GBR  *ITA* | FEMALE  FEMALE  *FEMALE* |
| **Statistical analysis** | S.A. Gomez | Statistician | COL | MALE |
| **Panel** | Y. Renard | Hernia surgeon | FRA | MALE |
|  | F. Kockerling | Hernia surgeon | GER | MALE |
|  | R. Fortelny | Hernia Surgeon | AUT | MALE |
|  | M.A. Garcia Urena | Hernia Surgeon | ESP | MALE |
|  | U. Bracale | General Surgeon | ITA | MALE |
|  | B. Romain | General Surgeon | FRA | MALE |
|  | C. Gaarder | Emergency Surgeon | NOR | FEMALE |
|  | J. Bullock | Patient partner | GBR | FEMALE |
|  | D. Cummings | Patient partner | GBR | MALE |
|  | S.A. Antoniou | Methodologist-General Surgeon | GRE | MALE |
| **External advisors** | H. Bougard | Hernia/Emergency Surgeon | SAF | FEMALE |
|  | J.A. Pereira | Hernia /Emergency | SPA | MALE |

**Author individual contribution according to CRediT taxonomy**

**C. Stabilini:** conceptualization, investigation, methodology, project administration, supervision, Writing original draft

**A. Theodorou:** data curation, investigation, writing original draft, Writing – review & editing

**E. Deerenberg:** data curation, investigation, writing original draft, Writing – review & editing

**M. Pawlak:** conceptualization, project administration, supervision, Writing original draft

**A.C. Quiroga Centeno:** data curation, investigation, Formal analysis, Writing – review & editing

**K. Gilmore:** data curation, investigation, Writing – review & editing

**S. Capoccia Giovannini:** data curation, investigation, formal analysis, visualization, Writing original draft

**F. Pecchini:** data curation, investigation, Writing – review & editing

**E. Schembari:** data curation, investigation, Writing – review & editing

**S.A. Gomez:** formal analysis, software

**Y. Renard:** validation, Writing – review & editing

**F. Kockerling:** validation, Writing – review & editing

**R. Fortelny:** validation, Writing – review & editing

**M.A. Garcia Urena:** validation, Writing – review & editing

**U. Bracale:** data curation, validation, Writing – review & editing

**B. Romain:** data curation, validation, Writing – review & editing

**C. Gaarder:** validation, Writing – review & editing

**J. Bullock:** validation

**D. Cummings:** validation

**S.A. Antoniou:** methodology, Writing – review & editing

**H. Bougard:** validation, Writing – review & editing

**J.A. Pereira:** validation, Writing – review & editing

**F. Berrevoet**: external advisor, validation, Writing – review & editing

**E. Mäkäräinen:** external advisor, validation, Writing – review & editing

**S. Morales-Conde:** external advisor, validation, Writing – review & editing
