# Supplementary material for: EHS Guidelines on the Management of Primary Ventral and Incisional Hernias Under Emergency Conditions
Source: J Abdom Wall Surg. 2026 Mar 11;5:16228. doi: 10.3389/jaws.2026.16228 (PMC13044802; doi:10.3389/jaws.2026.16228)
Supplement: Supplementary file 11 [file Supplementaryfile14.docx]

**Supplementary file 14**

| **Summary of findigs KQ6** | | | | | | |
| --- | --- | --- | --- | --- | --- | --- |
| **Synthetic permanent mesh compared to other mesh type in the Mesh Based Repair of emergency primary ventral and incisional hernia** | | | | | | |
| Outcomes | **Anticipated absolute effects^*^** (95% CI) | | Relative effect (95% CI) | № of participants (studies) | Certainty of the evidence (GRADE) | Comments |
|  | **Risk with other mesh type** | **Risk with Synthetic permanent mesh** |  |  |  |  |
| Mortality | 14 per 1.000 | **10 per 1.000** (6 to 18) | **OR 0.75** (0.43 to 1.28) | 29755 (2 non-randomised studies) | ⨁⨁⨁◯ Moderate^a^ | Synthetic permanent mesh likely results in little to no difference in mortality. |
| SSI | 526 per 1.000 | **167 per 1.000** (53 to 427) | **OR 0.18** (0.05 to 0.67) | 173 (2 non-randomised studies) | ⨁⨁◯◯ Low^b,c^ | The evidence suggests synthetic permanent mesh reduces SSI. |
| Recurrence | 71 per 1.000 | **74 per 1.000** (7 to 492) | **OR 1.04** (0.09 to 12.57) | 41 (1 non-randomised study) | ⨁◯◯◯ Very low^c,d^ | Synthetic permanent mesh may increase/have little to no effect on recurrence but the evidence is very uncertain. |
| ***The risk in the intervention group** (and its 95% confidence interval) is based on the assumed risk in the comparison group and the **relative effect** of the intervention (and its 95% CI).  **CI:** confidence interval; **OR:** odds ratio | | | | | | |

#### Explanations

a. more than 75% of papers at moderate Rob maximum

b. 60% of studies at maximum moderate risk of bias

c. very low number of events, small sample size

d. the paper is at serious rob

mortality

recurrence

ssi
