# Supplementary material for: EHS Guidelines on the Management of Primary Ventral and Incisional Hernias Under Emergency Conditions
Source: J Abdom Wall Surg. 2026 Mar 11;5:16228. doi: 10.3389/jaws.2026.16228 (PMC13044802; doi:10.3389/jaws.2026.16228)
Supplement: Supplementary file 12 [file Supplementaryfile15.docx]

**Supplementary file 15**

| **Summary of findings KQ 7** | | | | | | |
| --- | --- | --- | --- | --- | --- | --- |
| **Laparoscopic approach compared to Open approach for the Mesh Based Repair of emergency primary ventral and incisional hernia** | | | | | | |
| Outcomes | **Anticipated absolute effects^*^** (95% CI) | | Relative effect (95% CI) | № of participants (studies) | Certainty of the evidence (GRADE) | Comments |
|  | **Risk with Open approach** | **Risk with laparoscopic approach** |  |  |  |  |
| Mortality | 32 per 1.000 | **17 per 1.000** (13 to 22) | **OR 0.52** (0.40 to 0.68) | 34450 (5 non-randomised studies) | ⨁⨁⨁◯ Moderate^a^ | Laparoscopic approach probably reduces mortality slightly. |
| SSI | 70 per 1.000 | **29 per 1.000** (24 to 34) | **OR 0.39** (0.33 to 0.46) | 41883 (7 non-randomised studies) | ⨁◯◯◯ Very low^b,c^ | Laparoscopic approach may reduce/have little to no effect on SSI but the evidence is very uncertain. |
| RECURRENCE | 12 per 1.000 | **9 per 1.000** (2 to 40) | **OR 0.74** (0.16 to 3.48) | 728 (2 non-randomised studies) | ⨁◯◯◯ Very low^c,d,e^ | The evidence is very uncertain about the effect of laparoscopic approach on RECURRENCE. |
| REOPERATION | 47 per 1.000 | **32 per 1.000** (27 to 39) | **OR 0.67** (0.55 to 0.81) | 26641 (6 non-randomised studies) | ⨁⨁◯◯ Low^c,f^ | Laparoscopic approach may result in little to no difference in REOPERATION. |
| ***The risk in the intervention group** (and its 95% confidence interval) is based on the assumed risk in the comparison group and the **relative effect** of the intervention (and its 95% CI).  **CI:** confidence interval; **OR:** odds ratio | | | | | | |

#### Explanations

a. 85% of papers were at max moderate RoB

b. 60% of papers were at max moderate RoB

c. I2 50%

d. 60% of papers were at least at serious RoB

e. small sample, small number of events

f. 50% of papers were at high risk of bias

mortality

Recurrence

reoperation

SSI
