# Supplementary material for: EHS Guidelines on the Management of Primary Ventral and Incisional Hernias Under Emergency Conditions
Source: J Abdom Wall Surg. 2026 Mar 11;5:16228. doi: 10.3389/jaws.2026.16228 (PMC13044802; doi:10.3389/jaws.2026.16228)
Supplement: Supplementary file 13 [file Supplementaryfile4.docx]

**Supplementary file 4- Search strings for EHS guidelines on emergency management of Ventral hernia and inclusion and exclusion criteria for data extraction**

**Search Strategy in PubMed MEDLINE**

(ventral hernia* or incisional hernia* or (hernia* adj3 (ventral or incisional))).ti. and ((emergen* or urgen* or complicat* or incarcerat* or strangulat*).ti,ab. or exp Intestinal Obstruction/ or exp Hernia, Incarcerated/ or exp Hernia, Strangulated/) and (exp Surgical Procedures, Operative/ or surg*.ti. or repair*.ti. or herniorrhaphy.ti. or treatment*.ti. or management*.ti. or reconstruction.ti.)

**Search Strategy in SCOPUS**

( TITLE-ABS-KEY ( defect OR hernia ) ) AND ( TITLE-ABS-KEY ( ventral OR incisional ) ) AND ( TITLE-ABS-KEY ( emergent OR emergency OR urgent OR urgency OR complicated OR strangulated OR strangulation OR incarcerated ) ) AND ( TITLE-ABS-KEY ( repair OR herniorrhaphy OR reconstruction OR surgery OR surgical OR management OR treatment OR closure ) ) AND PUBYEAR > 1979 AND PUBYEAR < 2025 AND ( LIMIT-TO ( SUBJAREA , "MEDI" ) ) AND ( LIMIT-TO ( DOCTYPE , "ar" ) OR LIMIT-TO ( DOCTYPE , "re" ) OR LIMIT-TO ( DOCTYPE , "cp" ) ) AND ( LIMIT-TO ( LANGUAGE , "English" ) OR LIMIT-TO ( LANGUAGE , "Spanish" ) OR LIMIT-TO ( LANGUAGE , "Italian" ) OR LIMIT-TO ( LANGUAGE , "German" ) )

This supplementary file outlines the inclusion and exclusion criteria applied by the Evidence Review Team for study selection.

*Inclusion Criteria:*

- Study Design: Randomized controlled trials (RCTs) and observational comparative-cohort studies.
- Patient Population: Adult patients.
- Condition: Ventral hernia (including both incisional and primary ventral hernias) presenting under emergency conditions.

*Exclusion Criteria*:

- Study Design: Non-comparative studies.
- Hernia Type: Inguinal or groin defects, parastomal hernia.
- Clinical Scenario: Open abdomen or burst abdomen closure, non-emergency conditions.
- Surgical Approach: Robotic procedures.

Articles were included in the analysis if extractable data specifically for patients undergoing emergency ventral hernia repair were available from cohorts that also included other hernia types or presentations. Otherwise, such articles were excluded
