# Supplementary material for: EHS Guidelines on the Management of Primary Ventral and Incisional Hernias Under Emergency Conditions
Source: J Abdom Wall Surg. 2026 Mar 11;5:16228. doi: 10.3389/jaws.2026.16228 (PMC13044802; doi:10.3389/jaws.2026.16228)
Supplement: Supplementary file 14 [file Supplementaryfile12.docx]

**Supplementary file 12**

| **Summary of findings KQ4a** | | | | | | |
| --- | --- | --- | --- | --- | --- | --- |
| **Mesh based repair compared to Open Abdomen in emergency treatment of primary ventral and incisional hernia with defects not amenable to closure in CDC ≥2 wound class** | | | | | | |
| Outcomes | **Anticipated absolute effects^*^** (95% CI) | | Relative effect (95% CI) | № of participants (studies) | Certainty of the evidence (GRADE) | Comments |
|  | **Risk with Open Abdomen** | **Risk with Closure of the abdominal Cavity** |  |  |  |  |
| Morbidity (Clavien Dindo >3b) | 750 per 1.000 | **351 per 1.000** (130 to 677) | **OR 0.18** (0.05 to 0.70) | 40 (1 non-randomised study) | ⨁◯◯◯ Very low^a,b,c^ | Closure of abdominal cavity may results in a reduction in CD >3B. |
| REOPERATION | 800 per 1.000 | **242 per 1.000** (74 to 597) | **OR 0.08** (0.02 to 0.37) | 40 (1 non-randomised study) | ⨁◯◯◯ Very low^a,b,c^ | Closure of abdominal cavity may reduce REOPERATION. |
| MORTALITY | 300 per 1.000 | **168 per 1.000** (45 to 458) | **OR 0.47** (0.11 to 1.97) | 44 (1 non-randomised study) | ⨁◯◯◯ Very low^a,b,c^ | Closure of abdominal cavity may results in little to no difference in MORTALITY. |
| ***The risk in the intervention group** (and its 95% confidence interval) is based on the assumed risk in the comparison group and the **relative effect** of the intervention (and its 95% CI).  **CI:** confidence interval; **OR:** odds ratio | | | | | | |

#### Explanations

a. ROBINS-I evaluation granted a serious Risk of Bias (see appendices)

b. cohort of patients not affected by an IH, nevertheless the condition treated correspond to the KQ formulated

c. limited sample size

**mortality**

**reoperation**

**morbidity**
